# Effects of dispersal and temperature variability on phytoplankton realized temperature niches

**DOI:** 10.1002/ece3.10882

**Published:** 2024-02-07

**Authors:** Alaina N. Smith, Andrew D. Barton

**Affiliations:** ^1^ Scripps Institution of Oceanography University of California San Diego La Jolla California USA; ^2^ Department of Ecology, Behavior and Evolution University of California San Diego La Jolla California USA

**Keywords:** mass effects, metacommunity, phytoplankton, storage effects, temperature niches

## Abstract

Phytoplankton species exhibit fundamental temperature niches that drive observed species distributions linked to realized temperature niches. A recent analysis of field observations of *Prochlorococcus* showed that for all ecotypes, the realized niche was, on average, colder and wider than the fundamental niche. Using a simple trait‐based metacommunity model that resolves fundamental temperature niches for a range of competing phytoplankton, we ask how dispersal and local temperature variability influence species distributions and diversity, and whether these processes help explain the observed discrepancies between fundamental and realized niches for *Prochlorococcus*. We find that, independently, both dispersal and temperature variability increase realized temperature niche widths and local diversity. The combined effects result in high diversity and realized temperature niches that are consistently wider than fundamental temperature niches. These results have broad implications for understanding the drivers of phytoplankton biogeography as well as for refining species distribution models used to project how climate change impacts phytoplankton distributions.

## Graphical abstract

Using a simple trait‐based metacommunity model that resolves fundamental temperature niches for a range of competing phytoplankton, we ask how dispersal and local temperature variability influence species distributions and diversity. We find that, independently, both dispersal and temperature variability increase realized temperature niche widths and local diversity but the combined effects result in high diversity and realized temperature niches that are consistently wider than fundamental temperature niches. These results have broad implications for understanding the drivers of phytoplankton biogeography as well as for refining species distribution models used to project how climate change impacts phytoplankton distributions.

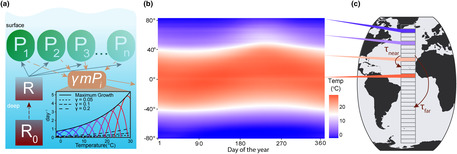

## 1 INTRODUCTION

Marine phytoplankton account for nearly 50% of global net primary production (Field et al., 1998) and drive important global biogeochemical cycles, such as the export of carbon from the ocean surface to depth (Falkowski et al., 1998). The roles they play in these cycles are highly dependent upon community structure and biodiversity patterns (Beaugrand et al., 2010; Guidi et al., 2016). Phytoplankton community structure is controlled by a combination of bottom‐up factors, including but not limited to nutrient supply (Edwards et al., 2013; Sunda & Huntsman, 1995), light (Geider et al., 1998), and temperature (Boyd et al., 2013; Eppley, 1972; Thomas et al., 2012), top‐down pressures such as grazing (Calbet & Landry, 2004; Ward et al., 2012), and factors that affect immigration and emigration (Villarino et al., 2018; Ward et al., 2021).

Temperature is an important factor that influences global phytoplankton distributions by impacting vital rates such as metabolism and growth (Eppley, 1972; Marañón et al., 2013) and by modulating local properties of the water column that influence the provision of nutrients and exposure of cells to light (Huisman et al., 2004). Collectively, the maximum possible specific growth rate across all species of phytoplankton increases exponentially with temperature (Eppley, 1972). However, each species of phytoplankton has a distinct thermal response curve, or fundamental temperature niche, defined by the range of temperatures where growth is positive (the niche width) and a temperature where growth is at its maximum (the optimal temperature) measured in laboratory conditions with no resource limitations or negative interactions (e.g., parasites, predators, or competition; Boyd et al., 2013; Marañón et al., 2013). These temperature preferences, in many cases, underlie observable phytoplankton biogeographic patterns across temperature gradients (e.g., Johnson et al., 2006; Thomas et al., 2012).

Biotic interactions such as competition and predation should, in theory, lead to narrower realized than fundamental temperature niches (Colwell & Rangel, 2009; Hutchinson, 1957). However, a recent study of *Prochlorococcus* temperature niches found that realized temperature niches were wider and colder than fundamental temperature niches, as measured in laboratory conditions, for four globally distributed ecotypes (Smith et al., 2021). *Prochlorococcus* is the most abundant photosynthetic microbe on Earth and is comprised of many ecotypes with distinct traits and biogeographies (Chisholm et al., 1992; Larkin et al., 2016; Rocap et al., 2003; Zinser et al., 2006). There are a range of mechanisms that may contribute to the observed differences between fundamental and realized temperature niches in *Prochlorococcus* ecotypes, including ecological interactions such as predation (Guillou et al., 2001), local adaptation (Martiny et al., 2019), or dispersal (Doblin & Van Sebille, 2016; Hellweger et al., 2016). Here, we examined how spatial mass effects and temporal storage effects—defined broadly as the occurrence of species in habitats where their net growth is negative, but the populations survive due to immigration or temporal persistence—are one possible explanation for the discrepancies between fundamental and realized temperature niches in *Prochlorococcus* ecotypes. Despite having negative net growth rates, the presence of a species is still ecologically important to community dynamics, the food web, and ecosystem functions. Spatial mass effects are the net flow of individuals between local patches driven by dispersal (Leibold et al., 2004; Shmida & Wilson, 1985; Zonneveld, 1995); we use “spatial mass effects” in this context instead of “spatial storage effects.” Temporal storage effects (also called temporal mass effects) describe the role that environmental fluctuation plays in supporting diversity by providing multiple windows of opportunity for species with different niche preferences to optimize growth (Cáceres, 1997; Ellner et al., 2016; Kelly & Bowler, 2005; Kremer & Klausmeier, 2017; Zonneveld, 1995).

We created a simple metacommunity model to test how spatial mass and temporal storage effects influence phytoplankton realized temperature niches and community diversity. The model simulates a latitudinal transect through the ocean where phytoplankton communities are connected via isotropic dispersal that decreases in strength with increasing distance, and the temperature seasonality at each latitude is tied to marine observations. We refer to this seasonal change in temperature as “temperature variability” hereafter, but recognize that shorter‐ (e.g., storms, internal waves, upwelling) and longer‐term variations (e.g., natural and anthropogenic climate change) are important but are not examined further. Model phytoplankton species each have a unique temperature niche, but equivalent affinities for light and nutrients and equivalent dispersal capacity. Using this idealized framework, and through a sequence of controlled model experiments varying temperature variability, dispersal, and phytoplankton mortality, we ask: (1) How does the rate of dispersal affect phytoplankton realized temperature niches and local community diversity?, (2) How does the degree of temperature variability affect phytoplankton realized temperature niches and local community diversity?, and (3) How does the strength of phytoplankton mortality modulate the effects of dispersal and temperature variability on realized temperature niches and community diversity? While our model is designed to mimic essential properties of phytoplankton in marine settings, it is general enough to have relevance to other types of metacommunities. The model helps understand how ubiquitous spatial mass and temporal storage effects in the ocean may play important roles in shaping realized niches and community diversity.

## 2 METHODS

### 2.1 Model description

**FIGURE 1:**
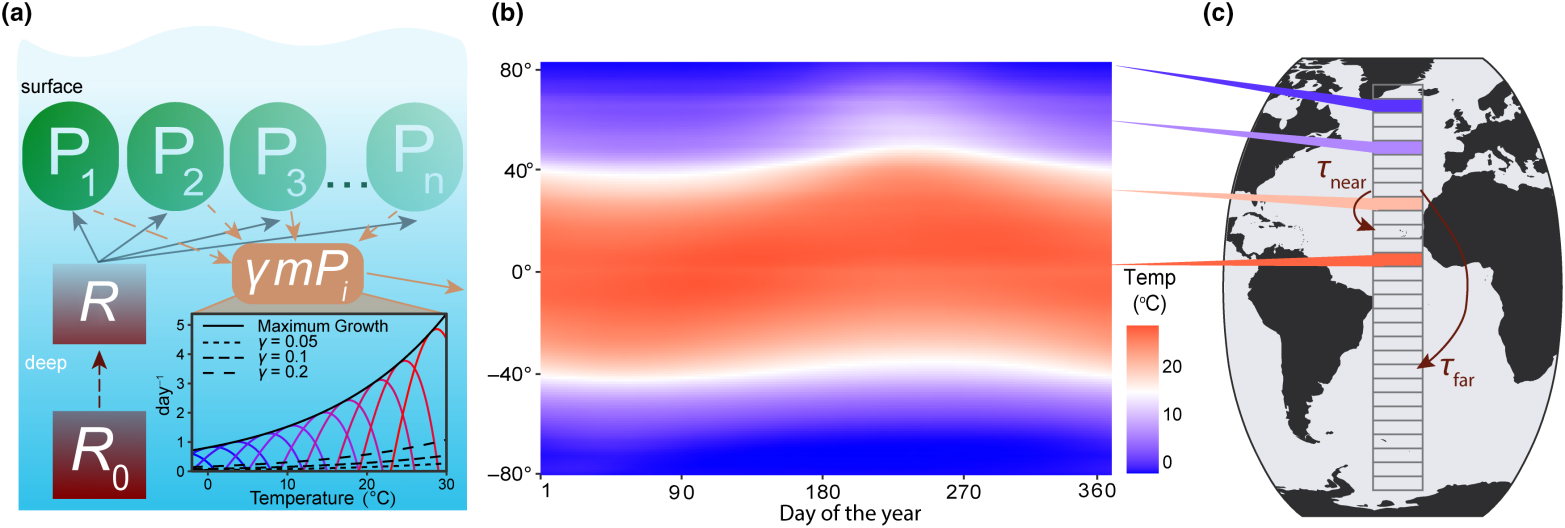
Schematic of the model. (a) The model resolves *n* different phytoplankton species (in this case 45), each with their own unique fundamental temperature niche (see (a) inset). The limiting resource (*R*) is supplied from deep water with a high and steady resource concentration (*R*_0_) at a constant rate (*d*; dark red dashed line). Phytoplankton loss is represented by a temperature‐dependent mortality (*m*(*T*)) term which is calculated as $\mathrm{\gamma a}e^{\mathrm{bT}}$ where $ae^{\mathrm{bT}}$ is the maximum growth rate as a function of temperature (solid exponential line in the inset), *a* and *b* are empirically‐determined constants, and γ which is a unitless scaling factor. We tested three different scaling factors (γ) to model high (γ = 0.2; long‐dash line), intermediate (γ = 0.1; short‐dash line), and low (γ = 0.05; dotted line) mortality pressures. (b) Seasonally varying sea surface temperature (SST) for each latitude in the model was derived from daily NOAA climatological SST data averaged across all longitudes and interpolated to the model timescale. (c) The latitudinal transect across the Atlantic Ocean was split into 159 different 1° latitude boxes in the model where the dispersal rate (τ) is a function of the distance between boxes and horizontal diffusivity. Boxes closer together (τ_near_) have stronger exchange rates compared to boxes further apart (τ_far_). The four boxes centered on 10°, 35°, 60°, and 75° (colored) are the four latitudes used in Figures 3, 4, 7, and 10 to demonstrate model output under different temperature conditions.

A latitudinal transect from 80° S to 80° N was divided into 159 1° latitude wide model boxes where each box is seeded with the same initial community comprised of 45 unique phytoplankton species (Figure 1). Model phytoplankton have equivalent affinity for nutrients but different temperature functional responses. The model does not consider light and how it impacts phytoplankton growth. The temperature conditions in each box are informed from sea surface temperature observations, and the dispersal rates between boxes are calculated based on estimated rates of horizontal eddy diffusivity in the ocean. Here, we outline the equations and assumptions used in the model for phytoplankton competition, nutrient supply, and dispersal between boxes.

Phytoplankton biomass (*P*; mmol P m^−3^) for each species (*i*) in each box (*j*) is controlled by the temperature‐dependent growth rate of the species ($\mu_{i,j}\left(T\right)$; day^−1^), the concentration of resources (*R_j_*; mmol P m^−3^), the temperature‐dependent mortality $m_{i,j}\left(T\right)$ (day^−1^) scaled by $\gamma \;\left(\text{unitless}\right)$, and immigration or emigration of species from and to adjacent boxes (i.e., net dispersal):

(1) $$\frac{dP_{i,j}}{\mathrm{dt}}=\mu_{i,j}\left(T\right)\frac{R_{j}}{R_{j}+k_{i}}P_{i,j}-\gamma m_{i,j}\left(T\right)P_{i,j}+\mathrm{net}\;{\text{dispersal}}_{i,j}$$

The resource concentration in each box is controlled by the influx of nutrients from a deep nutrient pool (*R*_0_ = 0.8 mmol P m^−3^) at a rate of *d* (0.864 day^−1^), representing a chemostat. The resource in each box is depleted by phytoplankton growth, which is represented by Michaelis–Menten nutrient uptake where all species have the same half‐saturation nutrient concentration (*k_i_*; mmol P m^−3^):

(2) $$\frac{dR_{j}}{\mathrm{dt}}=d\left(R_{0}-R_{j}\right)-\sum_{i}^{n}\left[\mu {\left(T\right)}_{i,j}\frac{R_{j}}{R_{j}+k_{i}}P_{i,j}\right]$$

Initially, all phytoplankton species begin with a concentration 10^−3^ mmol P m^−3^ and the resource concentration in each box starts at 10^−3^ mmol P m^−3^. Phytoplankton growth for each species in each box (here we drop the *j* subscript) is a function of temperature (Thomas et al., 2012):

(3) $$\mu_{i}\left(T\right)=ae^{\mathrm{bT}}\left[1-{\left(\frac{T-z_{i}}{\frac{w}{2}}\right)}^{2}\right]$$

where the constants *a* and *b* are empirically derived values that control the exponential increase of growth rate with temperature and the trait parameters (*z*_*i*_ and *w*) control the species‐specific response to temperature. The values for *a*, the growth rate at 0°C (0.81 day^−1^), and *b*, the exponential increase in growth rate with temperature (0.0631 day^−1^), are taken from empirical analyses (Bissinger et al., 2008) and are commonly utilized (Smith et al., 2021; Thomas et al., 2012). All model species have the same niche width (*w*; 10°C), which is roughly the niche width of observed North Atlantic phytoplankton species (Irwin et al., 2012). We created a vector of 45 unique $z_{i}$ values ranging from −4 to 40°C at 1°C increments and calculated a thermal growth curve with each $z_{i}$ across a gradient of temperatures between −4 and 50°C at 0.1°C increments. This gradient extends beyond realistic ranges in temperature to ensure full coverage. For each growth curve, we calculated the temperature where growth was maximum to define the optimal temperature (*T*_opt_; °C). Thus, each model phytoplankton species has a unique fundamental temperature niche and optimum temperature, but equivalent niche width (Figure 1a).

The rate of mortality ($m_{i}\left(T\right);{\mathrm{day}}^{-1}$) increases with temperature, similar to phytoplankton maximum growth rates:

(4) $$m_{i}\left(T\right)=ae^{\mathrm{bT}}$$

where *a* and *b* are the same constants in Equation (3). We test three different scaling factors (γ), 0.05, 0.1, and 0.2, in order to observe how low, intermediate, and high mortality influence the resulting realized temperature niches ($\gamma m_{i}\left(T\right)$; Figure 1a). Temperature‐dependent mortality has been used in other modeling studies (Thomas et al., 2012), and coarsely represents the increased growth rate of predators (e.g., Vidal, 1980) and phytoplankton respiration rates with increasing temperature (e.g., Brown et al., 2004).

Each model box had a climatological seasonal temperature cycle derived from daily sea surface temperature data from National Oceanic and Atmospheric Administration Optimum Interpolation SST data (NOAA OISST; https://www.ncei.noaa.gov/products/optimum‐interpolation‐sst; Reynolds et al., 2002). For each box, the daily temperature was averaged over all longitudes and averaged over 1982–2010, then interpolated to the model time step (Figure 1b). For model experiments with steady temperatures, we calculated the mean temperature from the seasonal cycle and set that as the constant temperature for the model box for each time point.

The rate of dispersal between any two model boxes (τ; day^−1^) is determined from horizontal eddy diffusivity ($K_{H}$; m^2^ s^−1^) in the ocean and the distance between boxes (∆y; m):

(5) $$\tau =\frac{K_{H}}{{\left(∆y\right)}^{2}}$$

$K_{H}$ ranges from 10^1^ to 10^4^ m^2^ s^−1^ in the ocean (Abernathey & Marshall, 2013), and we tested four different increments of $K_{H}$ for increasing diffusivity: 10^0^, 10^1^, 10^2^, and 10^3^ m^2^ s^−1^, excluding the high diffusivity values (10^4^ m^2^ s^−1^), as they are found in restricted areas, and including a very low diffusivity simulation (10^0^ m^2^ s^−1^). ∆y increases with the distance between boxes. τ increases with $K_{H}$ and decreases with ∆y, and was converted into units of day^−1^.

Net dispersal for each species *i* is the balance between immigration from all other boxes (there are *b* boxes) and emigration to all other boxes, such that net dispersal = immigration – emigration. For each species in box 1 (*j = 1*), net dispersal is:

(6) $$\begin{array}{c} \mathrm{net}\;{\text{dispersal}}_{i,j=1}={\text{immigration}}_{i,j=1}-{\text{emigration}}_{i,j=1}\\ \;=\sum_{j=2}^{b}\tau_{1,j}P_{i,j}-\sum_{j=2}^{b}\tau_{1,j}P_{i,j=1} \end{array}$$

where $\tau_{1,j}$ is the dispersal rate between box 1 and box *j*. Immigration depends upon the biomass of species *i* in box *j* ($P_{i,j}$), whereas emigration depends upon the biomass of species *i* in box 1 ($P_{i,j=1}$). Net dispersal for all other boxes was calculated in a similar manner.

Model simulations were run for 50 years with a time step of 3 h, and we present results averaged over the last 5 years of the model integration.

### 2.2 Estimating fundamental and realized niches

After 50 years, the model species in each box were categorized as extant if: (1) concentration in the last year of the model was greater than 10^−4^ times the maximum concentration for any given species and (2) the difference between yearly averages phytoplankton concentration was approximately zero for the last 5 years of the model ($\frac{\mathrm{dP}}{\mathrm{dt}}≅0$). Species not meeting these criteria were considered, in practical terms, extirpated. The realized temperature niche for each surviving species was calculated by fitting a nonparametric kernel density estimate to the biomass (*P*) as a function of temperature (*T*) for each species across all boxes. Using a kernel density estimate allowed for a continuous curve estimate over the finite range of temperatures in the model and made it simple to calculate the maximum and width of the curve without introducing bias via parameterization (Antell et al., 2021; Broennimann et al., 2012; Smith et al., 2021). From the curve fit, the modeled realized optimal temperature ($T_{R}^{\mathrm{opt}}$) and niche width (*W*_*R*_) were calculated as follows:

(7) $$T_{R}^{\mathrm{opt}}=T\left[P_{\max}\right]$$

(8) $$W_{R}=T_{0.99}-T_{0.01}$$

where the optimal temperature ($T_{R}^{\mathrm{opt}}$) is defined as the temperature (*T*) where biomass is at its max (*P*_max_), and the niche width (*W*_*R*_) is defined as the difference between the 1st and the 99th percentile of the temperature distribution. To compare the difference between the modeled and the fundamental niche widths, we calculated the following ratio:

(9) $$\delta_{W}=\frac{\left(W_{R}-W_{F}\right)}{W_{F}}$$

$W_{F}$ is equal to 10°C for all species in the model (Equation 3). A positive (negative) value of $\delta_{W}$ means that the modeled realized temperature niche is wider (narrower) than the fundamental temperature niche width.

To compare the difference between the modeled and the fundamental temperature optimums, we calculated the following ratio:

(10) $$\delta_{T^{\mathrm{opt}}}=\frac{\left(T_{R}^{\mathrm{opt}}-T_{F}^{\mathrm{opt}}\right)}{T_{F}^{\mathrm{opt}}}$$

$T_{F}^{\mathrm{opt}}$ varies across model species (see Equation 3). A positive (negative) value of $\delta_{T^{\mathrm{opt}}}$ means that the modeled realized temperature optimum is warmer (colder) than the fundamental temperature optimum.

### 2.3 Diversity metrics

Average diversity over the last 5 years of the model ($\bar{S}$) for each box was calculated by averaging the number of species present at each time point in the last 5 years of the model, using the two criteria outlined above. Total diversity (*S*_*T*_) in each box was calculated by summing the total number of unique species present at any time in the last 5 years of the model.

### 2.4 Model experiments

**FIGURE 2:**
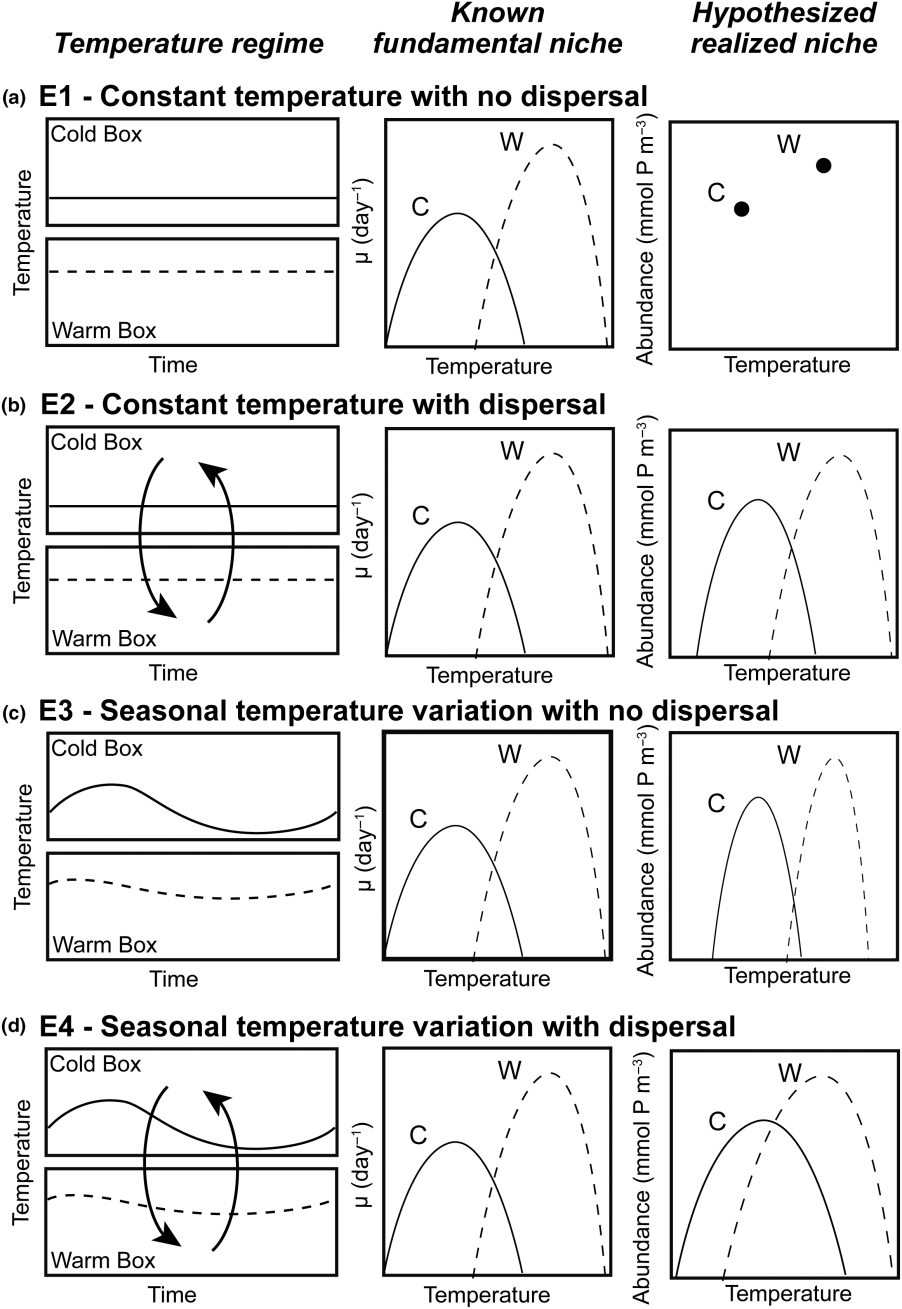
Schematic of model experiments (E1–E4). The left column demonstrates the yearly temperature cycle for cold (solid lines) and warm temperature boxes (dashed lines) for each experiment. Experiments with no temperature variability (E1 and E2) show constant temperature over time. Experiments with dispersal (E2 and E4) have arrows between boxes to represent dispersal between boxes. The middle and right columns show the fundamental niches and hypothesized realized niches for two species, one with a colder optimal temperature that would survive in the cold box (solid line) and one with a warmer optimal temperature that would be found in the warm box (dashed line). (a) Experiment 1 (E1) is run under constant temperature with no dispersal (control); (b) Experiment 2 (E2) is run under constant temperature with dispersal (spatial mass effects only); (c) Experiment 3 (E3) is run with seasonal temperature variation with no dispersal (temporal storage effects only); and (d) Experiment 4 (E4) is run with seasonal temperature variation with dispersal (spatial and temporal storage effects). The fundamental temperature niches are the same across all experiments (middle column), but the realized temperature niche (right column) is shaped by the temperature regime and dispersal. We represent the realized niche of the two species with points in E1 because the lack of temperature variability through time and without any exchange between boxes would not result in a measurable change in abundance over temperatures.

We conducted four model experiments, outlined in Figure 2, that tested ecological outcomes in phytoplankton community models run with: (E1) no spatial mass or temporal storage effects—a control experiment; (E2) spatial mass effects only; (E3) temporal storage effects only; and (E4) combined spatial mass and temporal storage effects.

In Experiment 1 (E1; Figure 2a), we implemented the phytoplankton community model with constant temperature in each box, determined by the mean temperature throughout the year at that location, and no dispersal. We hypothesized that competitive exclusion would lead to one dominant species present in each box, and the surviving species would be the one with the optimal temperature closest to that of the yearly average. We refer to this model experiment as a control.

In Experiment 2 (E2; Figure 2b), we kept a constant temperature in each box, as in E1, but allowed for model species to disperse between boxes, with varying dispersal strengths. We hypothesized that allowing for species to disperse between boxes would increase realized niche widths and diversity relative to the control experiment (E1; Figure 2a). Additionally, we hypothesized that as dispersal magnitude increases, realized temperature niche widths would increase beyond the fundamental temperature niche widths such that $\delta_{W}$ > 0 and species diversity within each box would increase.

In Experiment 3 (E3; Figure 2c), we allowed temperature within each box to vary seasonally, but did not allow for dispersal between boxes. The seasonal cycle of temperature within each box was tied to observations. Relative to the control experiment (E1; Figure 2a), we hypothesized that allowing for temperature to vary seasonally would increase realized niche widths and increase diversity. Additionally, we hypothesized that boxes with larger temperature amplitudes would support a greater number of species and have species with wider realized temperature niches relative to boxes with lower temperature amplitudes.

In Experiment 4 (E4; Figure 2d), we allowed model temperature to vary seasonally in each box and for species to disperse between boxes. We hypothesized that the combined influence of increasing dispersal and temperature variability would result in realized temperature niches that were wider than fundamental temperature niches ($\delta_{W}$ > 0) and would result in the highest diversity across all experiments.

In E2–E4, we explored model sensitivity to changing the strength of phytoplankton mortality (*γ*) and horizontal dispersal (τ). We explored the sensitivity of model results to the strength of phytoplankton mortality because it influences phytoplankton net growth rate and consequently competitive dynamics within each box. Additionally, horizontal diffusivity influences the rate and magnitude of immigration and emigration within a community influencing the overall competitive dynamics within each box.

## 3 RESULTS

### 3.1 (E1) control experiment with no mass effects

**FIGURE 3:**
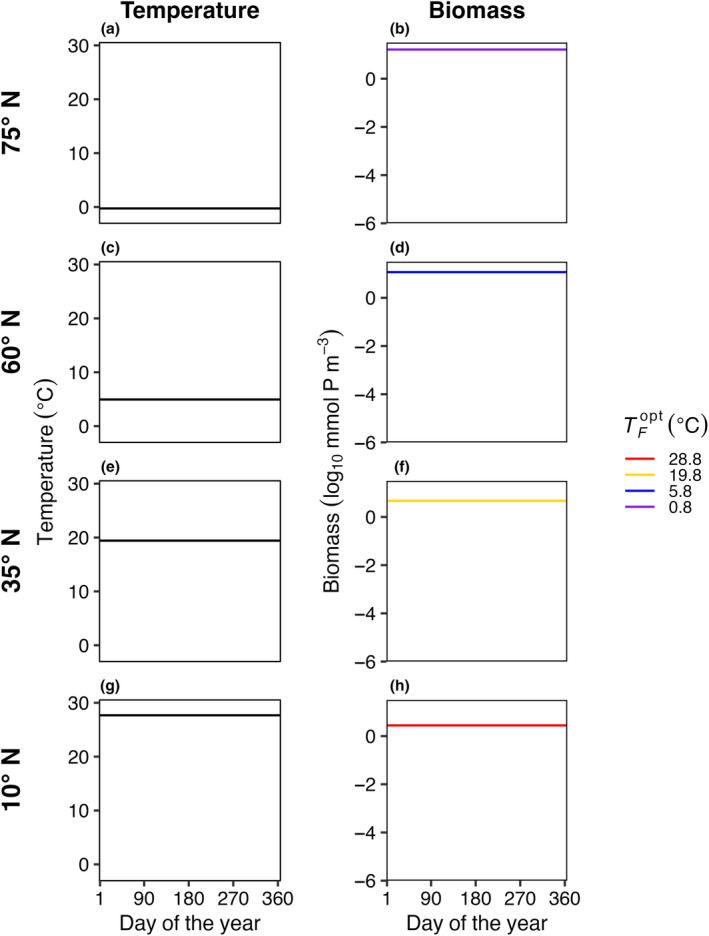
E1. Average daily temperature (left column: a, c, e, g) and biomass for surviving phytoplankton species (right column: b, d, f, h) in four illustrative model boxes (rows) ranging from colder high latitudes (top) to warmer low latitudes (bottom) in the last 5 years of a 50‐year model integration. The four illustrative model boxes represented areas centered on 75° N (a, b), 60° N (c, d), 35° N (e, f), and 10° N (g, h), as indicated in Figure 1c. Temperature (°C) in each illustrative box was constant through time but different between boxes. The surviving species (only one per box in this case) are colored by optimum temperature for growth ($T_{F}^{\mathrm{opt}}$).

In E1, model temperature was constant, there was no dispersal between boxes, and we test three mortality scaling factors (*γ* = 0.05, 0.1, and 0.2). In this case, only one species survived in each model box (Figure 3). The species with an optimal temperature ($T_{F}^{\mathrm{opt}}$) closest to the mean temperature of the environment had the highest net growth rate and outcompeted all other species. In this experiment, it was not possible to calculate a realized temperature niche for each species, as each species survived in only one box with one average temperature.

### 3.2 (E2) spatial mass effects only

**FIGURE 4:**
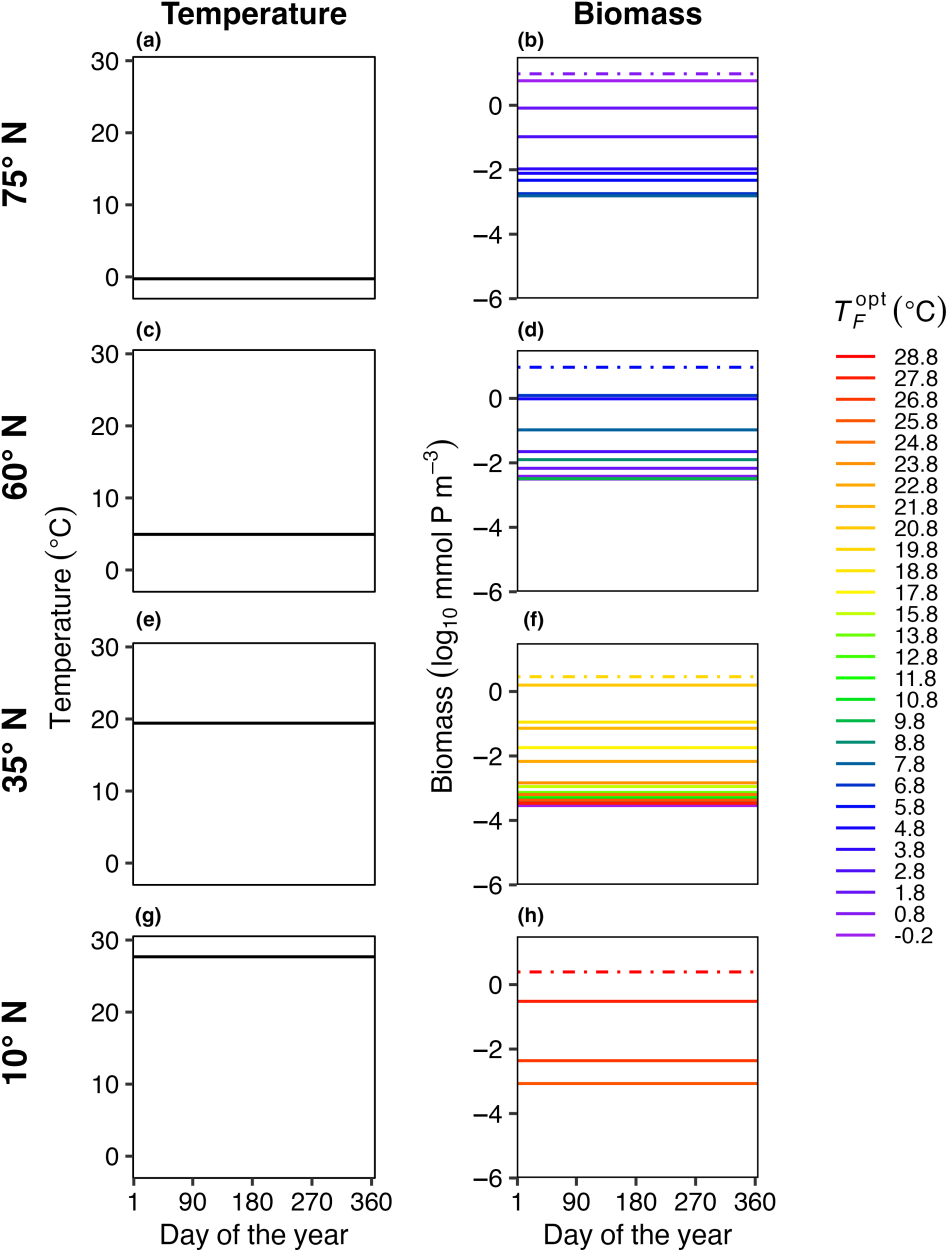
E2. Average daily temperature (left column: a, c, e, g) and biomass for surviving phytoplankton species (right column: b, d, f, h) in four illustrative model boxes (rows) ranging from colder high latitudes (top) to warmer low latitudes (bottom) in the last 5 years of a 50‐year model integration. The four illustrative model boxes represented areas centered on 75° N (a, b), 60° N (c, d), 35° N (e, f), and 10° N (g, h), as indicated in Figure 1c. Temperature (°C) in each illustrative box was constant through time but different between boxes. The surviving species are colored by their corresponding optimum temperature for growth ($T_{F}^{\mathrm{opt}}$). The dash‐dotted lines represent the species that were present in each box with constant temperature and no dispersal (Figure 3). Dispersal magnitude (*K*_*H*_) for this model run was 10^2^ m^2^ s^−1^ and the mortality scaling constant (γ) is equal to 0.05.

In E2, model temperature was constant, but phytoplankton dispersed between boxes. We also explored a range of dispersal strengths (τ) and phytoplankton mortality scaling factors (*γ*) in order to examine the effects of these mechanisms on model communities. Dispersal increased the number of species present in each box compared to the control (E1) with no dispersal (Figure 4); in this illustrative example, dispersal magnitude (*K*_*H*_) was 10^2^ m^2^ s^−1^ and the mortality scaling constant (γ) was 0.05. Rather than only one species being present per box, as in E1, typically four to ten species were present in E2 per model box, due to an influx of phytoplankton from neighboring model areas with different average but constant temperatures. Even with dispersal, the model phytoplankton with an optimum temperature closest to the average temperature in that box was the most abundant (dotted lines in Figure 4).

**FIGURE 5:**
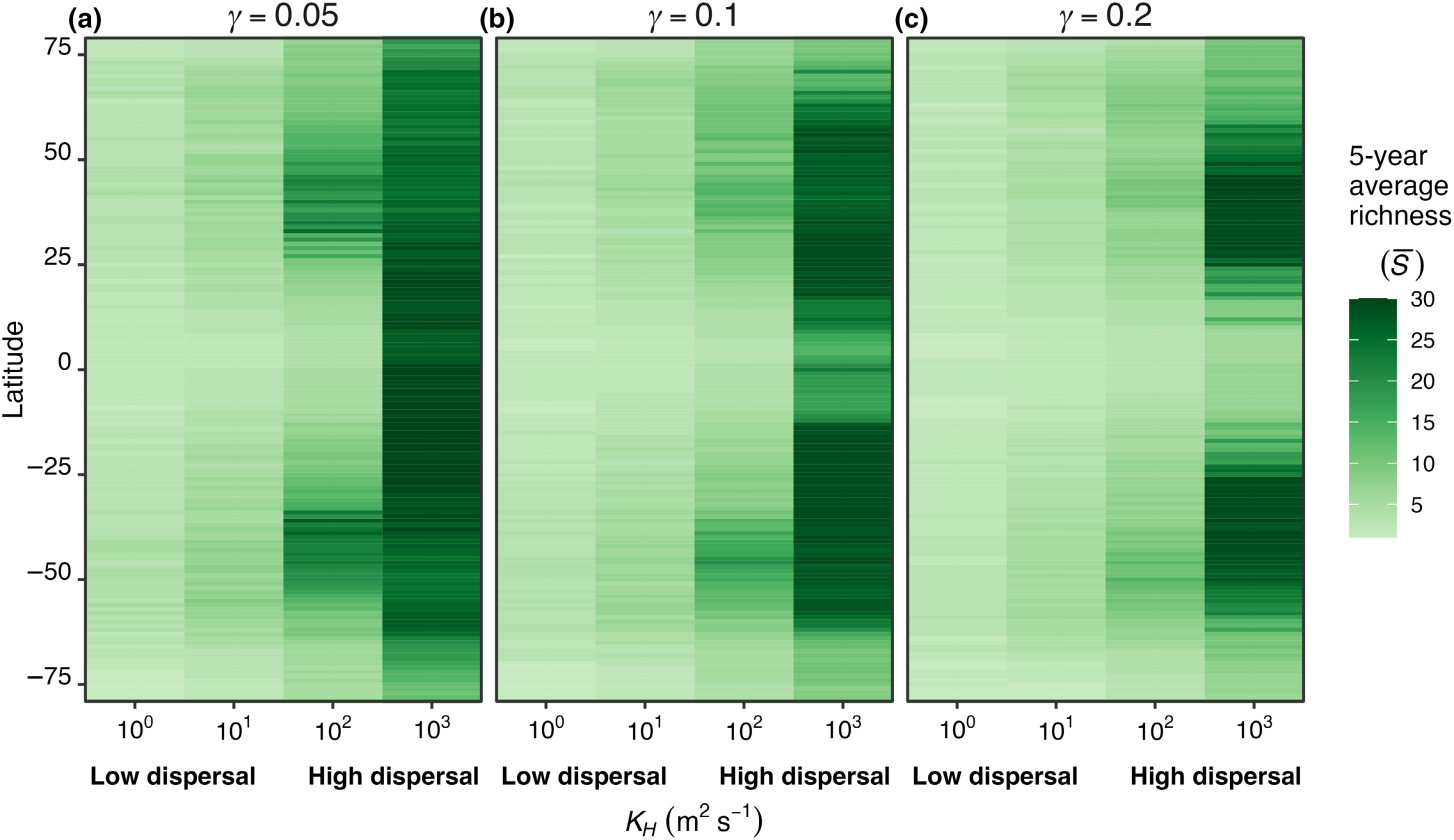
E2. Average model phytoplankton diversity ($\bar{S}$) at each model box latitude (*Y*‐axis) and for a range of dispersal strengths (*X*‐axis) for (a) low (γ = 0.05), (b) medium (γ = 0.1), and (c) high (γ = 0.2) mortality scaling factors (γ). $\bar{S}$ is the average number of species present at any time point during the last 5 years of a 50‐year model integration. Dispersal strength scaled with the horizontal diffusivity in the ocean (*K*_*H*_) which we varied from 10^0^ (low dispersal) to 10^3^ m^2^ s^−1^ (high dispersal).

The average number of model species surviving in each box, or $\bar{S}$, increased with dispersal magnitude (Figure 5). When the mortality rate was lowest (γ = 0.05; Figure 5a), with low dispersal (*K*_*H*_ = 10^0^ m^2^ s^−1^), the modeled latitudinal gradient in $\bar{S}$ was weak and $\bar{S}$ in each box was low. At the highest tested rate of dispersal (*K*_*H*_ = 10^3^ m^2^ s^−1^), $\bar{S}$ was high but nearly uniform across the boxes, meaning all species were dispersed rapidly enough to be present everywhere (Figure 5a). At intermediate levels of dispersal, $\bar{S}$ was greatest in mid‐latitudes due to the accumulation of both warm‐ and cold‐adapted species in these areas. Higher phytoplankton mortality (Figure 5b,c) yielded qualitatively similar changes in $\bar{S}$ with latitude and dispersal strength, except that the ubiquity of high $\bar{S}$ across latitude as in Figure 5a was not found and diversity was concentrated in the mid‐latitudes.

**FIGURE 6:**
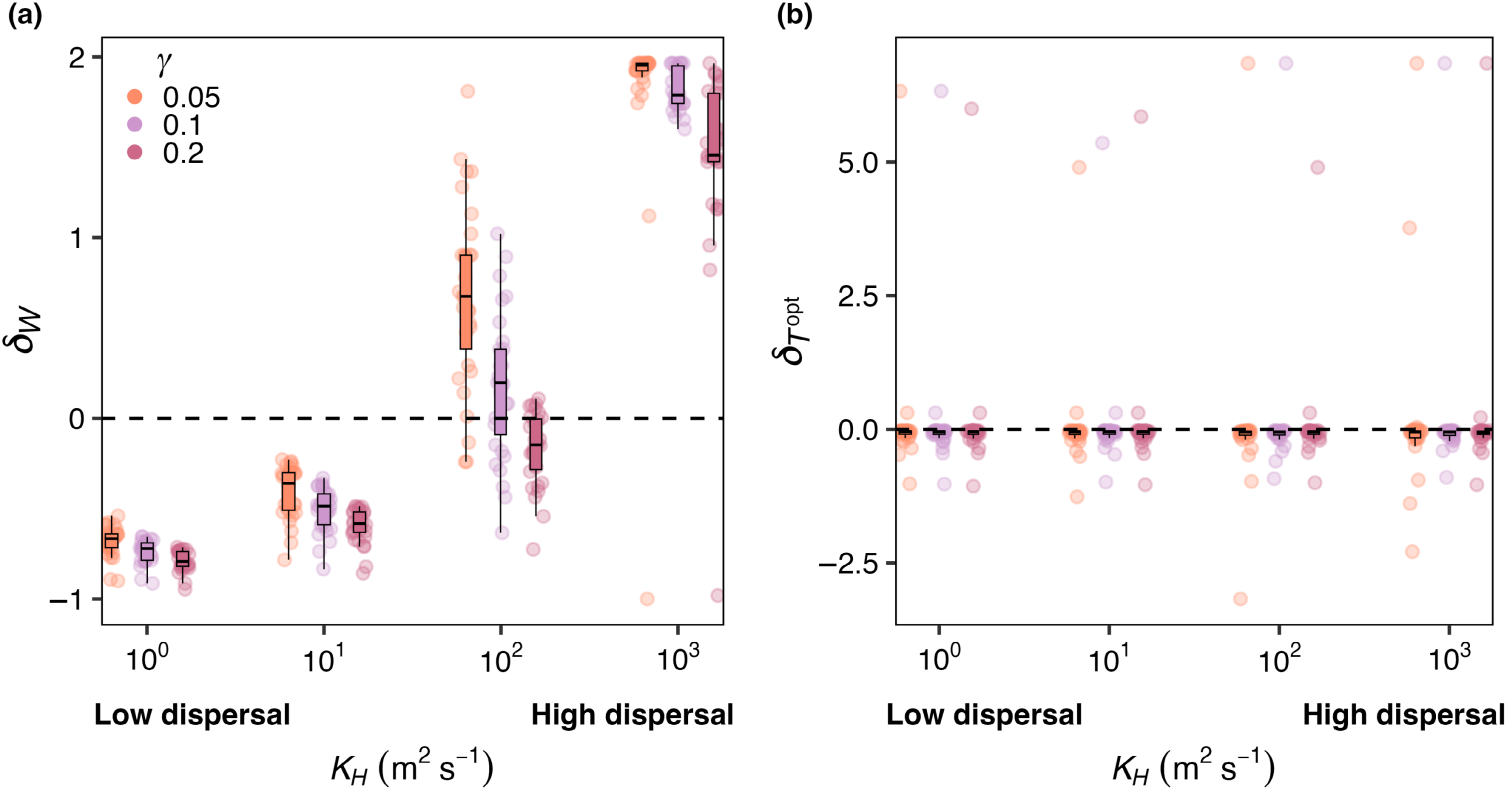
E2. (a) $\delta_{W}$ and (b) $\delta_{T^{\mathrm{opt}}}$ for a range of dispersal strengths (*K*_*H*_) from low (10^0^ m^2^ s^−1^) to high dispersal (10^3^ m^2^ s^−1^) and phytoplankton mortality scaling constants (γ). $\delta_{W}$ is the difference between realized and fundamental niche widths, divided by the fundamental niche width (Equation 9). $\delta_{T^{\mathrm{opt}}}$ is the difference between realized and fundamental temperature optimums, divided by the fundamental temperature optimum (Equation 10). *Y*‐axis values greater than zero mean the realized niche parameter was greater than the fundamental niche parameter. Colors represent low (orange; 0.05), medium (purple; 0.1), and high (pink; 0.2) phytoplankton mortality scaling constants (γ). Each circle within dispersal and mortality combinations represents one model species. The points are jittered along the *X*‐axis to better visualize variations in the *Y*‐axis. Overlaid on the raw data are boxplots to better visualize the differences between model parameter choices. The box represents the 25th and 75th percentile (bottom and top edges), and the 50th percentile (middle line). The lines are ±1.5 times the interquartile range, estimating the 95% confidence interval. Results are averaged over the last five years of a 50‐year model simulation.

Across all mortality scaling strengths (γ = 0.05, 0.1, and 0.2), realized temperature niche width ($W_{R}$) increased with increasing dispersal strength, but temperature niche optimums ($T_{R}^{\mathrm{opt}}$) were unaffected (Figure 6). When dispersal was low, realized temperature niche widths were consistently narrower than fundamental temperature niche widths (*δ*_*W*_ < 0; Figure 6a). Across all mortality scaling factors, $\delta_{W}$ increased as dispersal magnitude increased. On average, $\delta_{T^{\mathrm{opt}}}$ values were negative across all mortality strengths and dispersal magnitudes, meaning the realized temperature optimums were colder than the fundamental temperature optimums and varying dispersal and mortality did not change this (Figure 6b). Polar species with optimal temperatures close to or less than zero that thrive in the most extreme boxes end up with high $\delta_{T^{\mathrm{opt}}}$ values as they unilaterally disperse to adjacent boxes warmer temperatures thus increasing their realized temperature (Figure 6b).

### 3.3 (E3) temporal storage effects only

**FIGURE 7:**
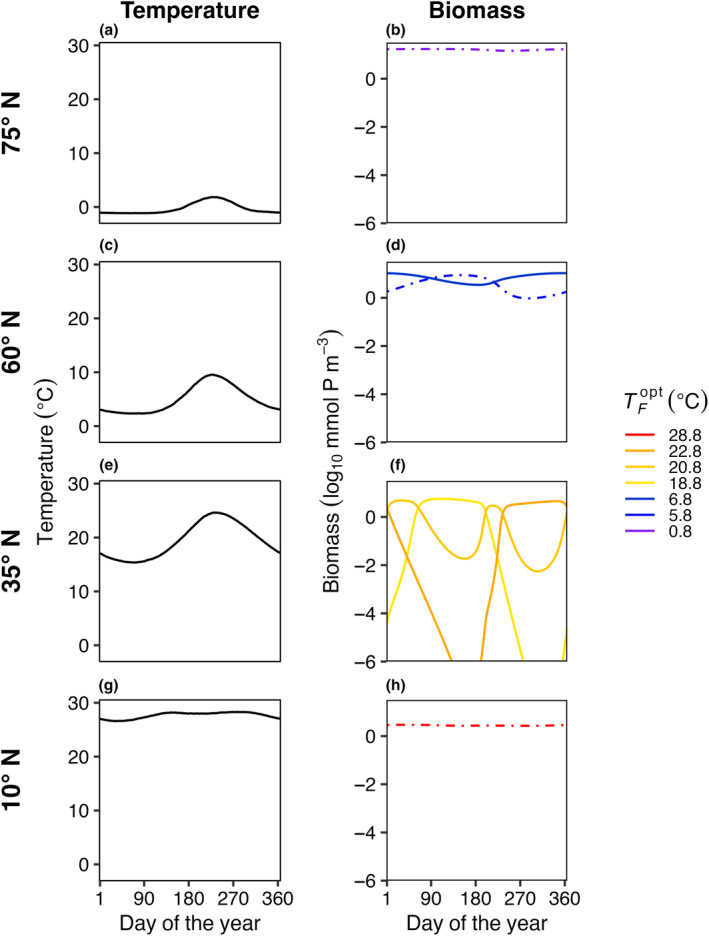
E3. Average daily temperature (left column: a, c, e, g) and biomass for surviving phytoplankton species (right column: b, d, f, h) in four illustrative model boxes in the last 5 years of a 50‐year model integration. The four illustrative model boxes (rows) ranging from colder high latitudes (top) to warmer low latitudes (bottom) represented areas centered on 75° N (a, b), 60° N (c, d), 35° N (e, f), and 10° N (g, h), as indicated in Figure 1c. Temperature (°C) in each illustrative box varied seasonally and differed between boxes. The surviving species are colored by their corresponding $T_{F}^{\mathrm{opt}}$ values. The dash‐dotted lines represent the species that were present in each box with constant temperature and no dispersal (Figure 3). Dispersal for this model run was zero and the phytoplankton mortality scaling factor (*γ*) was 0.05.

In E3, model temperature followed a box‐specific seasonal cycle, but phytoplankton were not able to disperse between boxes. Boxes with high seasonal temperature amplitudes (Figure 7c–f) had a greater number of species present compared to the control with no temperature variability (Figure 3). As a result of temperature varying seasonally, phytoplankton biomass was no longer constant over a model year and when more than one species is present, the community dynamics had a cyclical pattern where species increased and decreased in abundance following changes in temperature. The model phytoplankton species with an optimum temperature closest to the average temperature in that box was typically one of the most abundant species (dotted lines in Figure 7).

**FIGURE 8:**
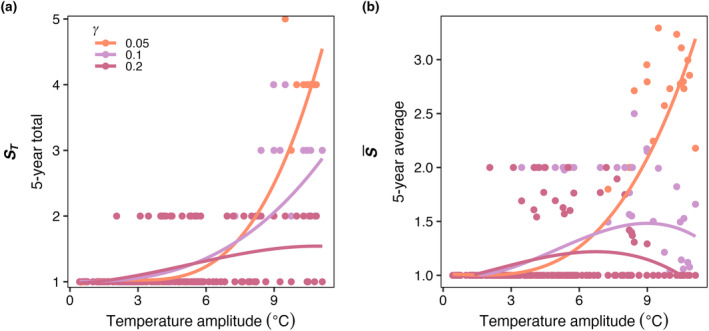
E3. (a) $\bar{S}$ and (b) *S*_*T*_ as a function of temperature amplitude (°C) in each box, for low (orange; 0.05), medium (purple; 0.1), and high (pink; 0.2) phytoplankton mortality scaling constants (γ). Each point represents the diversity metric for a single box plotted against the temperature amplitude of that box. $\bar{S}$ is the average number of species present at any point during the last 5 years of a 50‐year integration of the model, while *S*_*T*_ is the total number of species present during the last 5 years. Dispersal in this case was zero. The lines represent a GAM fit to visualize the change in diversity as temperature amplitude increases.

Compared to the control experiment with steady temperature (E1), seasonally varying temperature increased both the average number of species present ($\bar{S}$) and the total number of species present (*S*_*T*_) in the last 5 years of a 50‐year model integration across all phytoplankton mortality strengths (Figure 8). As temperature amplitude increased, the number of species present in the model increased when mortality was low (γ = 0.05). As the mortality scaling factor increased, the average number of species present decreased compared to low mortality (Figure 8a). The total number of species present (*S*_*T*_) increased as temperature seasonal variability increased and was similarly dampened by an increase in the mortality scaling factor relationship (Figure 8b).

**FIGURE 9:**
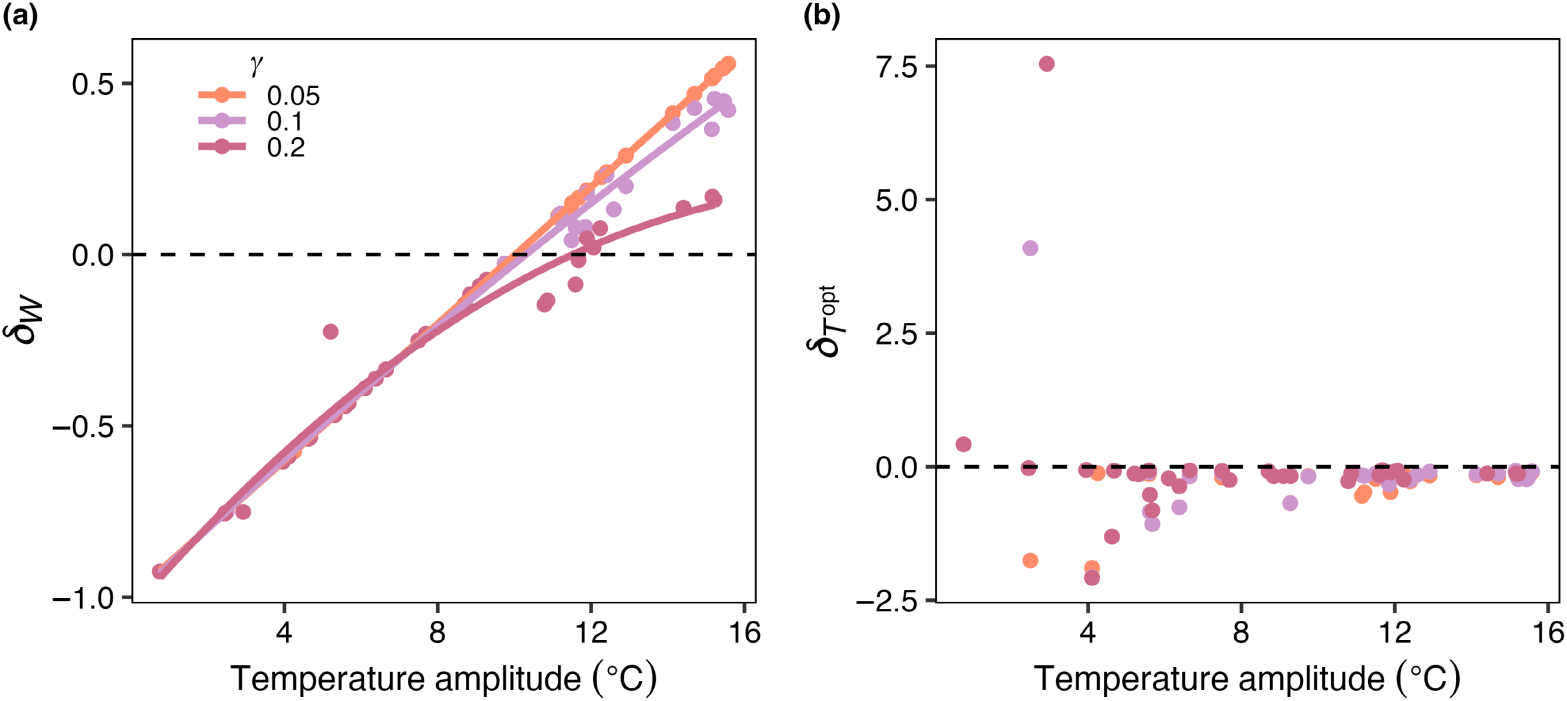
E3. (a) $\delta_{W}$ and (b) $\delta_{T^{\mathrm{opt}}}$ as function of temperature amplitude (°C), for low (orange; 0.05), medium (purple; 0.1), and high (pink; 0.2) phytoplankton mortality scaling constants (γ). $\delta_{W}$ is the difference between realized and fundamental niche widths, divided by the fundamental niche width (Equation 9). $\delta_{T^{\mathrm{opt}}}$ is the difference between realized and fundamental temperature optimums, divided by the fundamental temperature optimum (Equation 10). Points are realized niche parameter values for each species in the model calculated across all boxes where that species was present and considered present. Dispersal in this case was zero. Results are from the last 5 years of a 50‐year integration of the model. The points in (a) were fit with a GAM to illustrate the relationships between temperature amplitude and $\delta_{W}$.

Increasing temperature amplitude increased the realized temperature niche widths of the model phytoplankton species (Figure 9a) but had no effect on realized temperature optimums (Figure 9b). As mortality increased, there was a slight decrease in $\delta_{W}$, particularly when temperature amplitudes were high (Figure 9a), but there was no influence of mortality on $\delta_{T^{\mathrm{opt}}}$ (Figure 9b).

### 3.4 (E4) spatial mass and temporal storage effects

**FIGURE 10:**
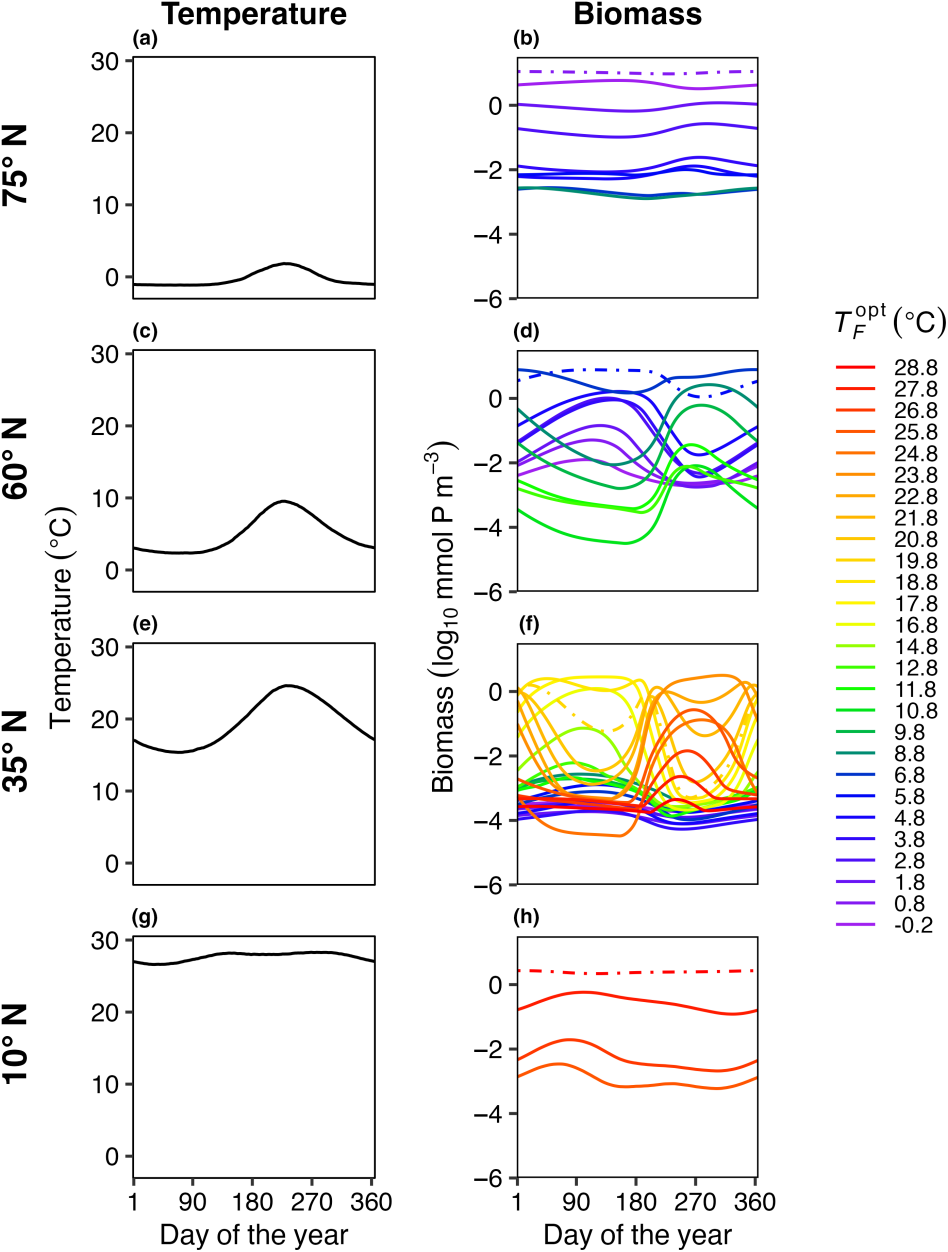
E4. Average daily temperature (left column: a, c, e, g) and biomass for surviving phytoplankton species (right column: b, d, f, h) in four illustrative model boxes (rows) ranging from colder high latitudes (top) to warmer low latitudes (bottom) in the last 5 years of a 50‐year model integration. The four illustrative model boxes represented areas centered on 75° N (a, b), 60° N (c, d), 35° N (e, f), and 10° N (g, h), as indicated in Figure 1c. Temperature (°C) in each illustrative box varied seasonally and differed between boxes. The surviving species are colored by their corresponding optimal temperature ($T_{F}^{\mathrm{opt}}$) values. The dash‐dotted lines represent the species that were present in each box with constant temperature and no dispersal (E1; Figure 3). Dispersal magnitude (*K*_*H*_) for this model run was 10^2^ m^2^ s^−1^ and the mortality scaling constant (γ) was 0.05.

In E4, the model temperature followed a box‐specific seasonal cycle and phytoplankton dispersed between boxes. Seasonal temperature variability and dispersal promoted greater species diversity in the model when compared to other model experiments. Boxes with high seasonal variability (Figure 10c–f) supported more species compared to boxes with low temperature variability (Figure 10a,b,g,h). However, as a result of the combined effects of temperature variability and dispersal, all boxes supported a greater number of species compared to previous experiments when the model was driven solely by either temperature variability or dispersal (Figures 4 and 7).

**FIGURE 11:**
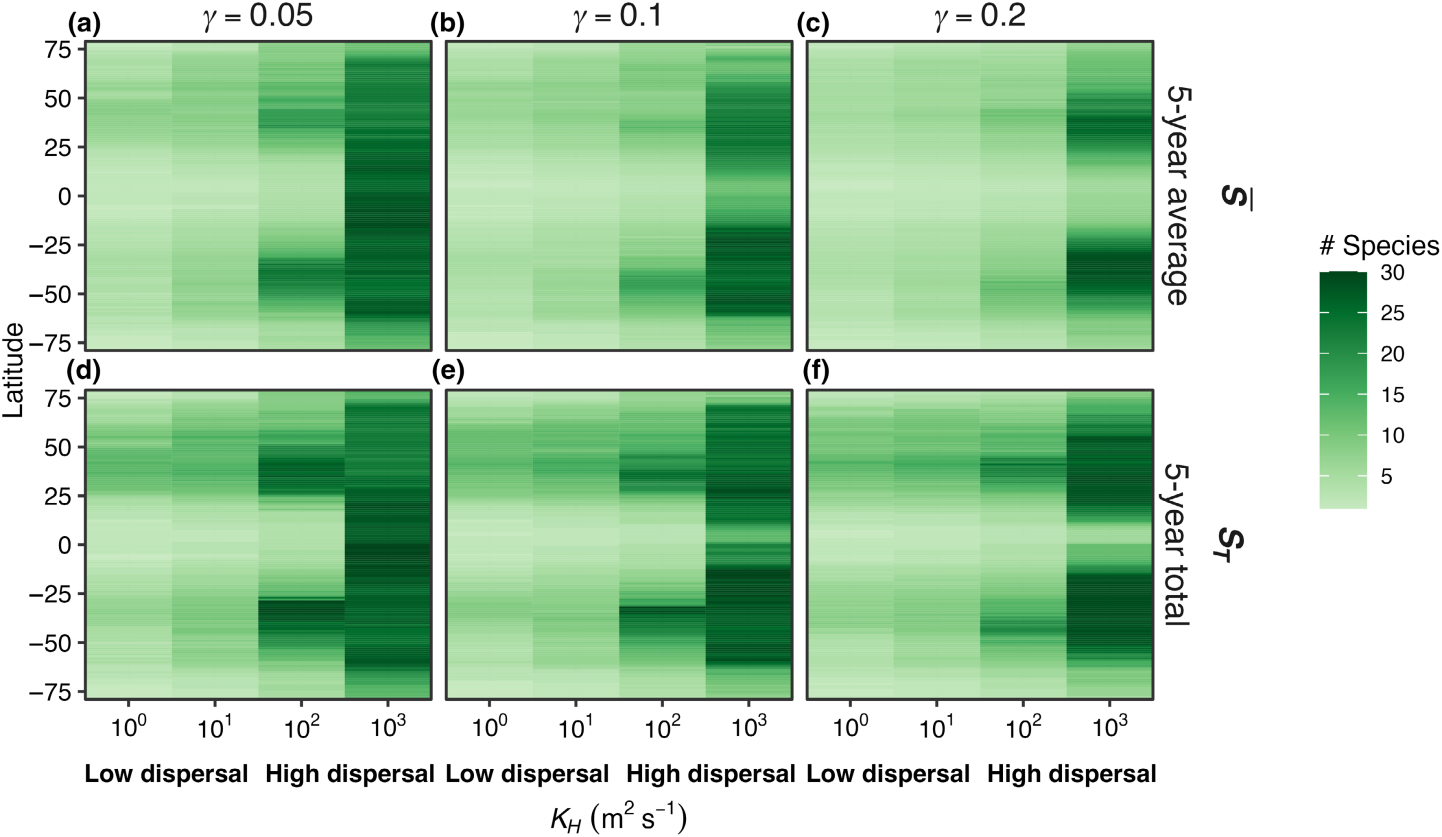
E4. (a–c) $\bar{S}$ and (d–f) *S*_*T*_ across latitude (*Y*‐axis) and across increasing dispersal strength (*X*‐axis) for low (0.05), medium (0.1), and high (0.2) phytoplankton mortality scaling factors (γ), respectively. $\bar{S}$ is the average number of species present at any point during the last 5 years of a 50‐year integration of the model, while *S*_*T*_ is the total number of species present during the last 5 years. Dispersal strength scaled with the horizontal diffusivity in the ocean (*K*_*H*_) which we varied from 10^0^ (low dispersal) to 10^3^ (high dispersal) m^2^ s^−1^.

As dispersal magnitude and seasonal temperature amplitude increased, the average number of model species present in each box ($\bar{S}$) and the total number of species present at any time in the last 5 years of the 50‐year model run (*S*_*T*_) increased (Figure 11). When temperature is constant but dispersal increases to 10^2^ m^2^ s^−1^ (E2), the maximum $\bar{S}$ (Figure 5a) and *S*_*T*_ (Figure 5b) values were 27, 20, and 14 species with low (γ = 0.05), medium (γ = 0.1), high (γ = 0.2) mortality scaling factors. When there was no dispersal but seasonally variable temperature (E3), the maximum $\bar{S}$ (Figure 8a) values were 3.29, 2.59, and 2 species; and the maximum S_T_ values across any box was 5, 4, and 2 species (Figure 8b) with low (γ = 0.05), medium (γ = 0.1), high (γ = 0.2) mortality scaling factors. Combining the effect of dispersal and temperature variability (E4) increased the maximum $\bar{S}$ and *S*_*T*_ values to 30, 30, and 25 species with low (γ = 0.05), medium (γ = 0.1), high (γ = 0.2) mortality scaling factors (Figure 11).

**FIGURE 12:**
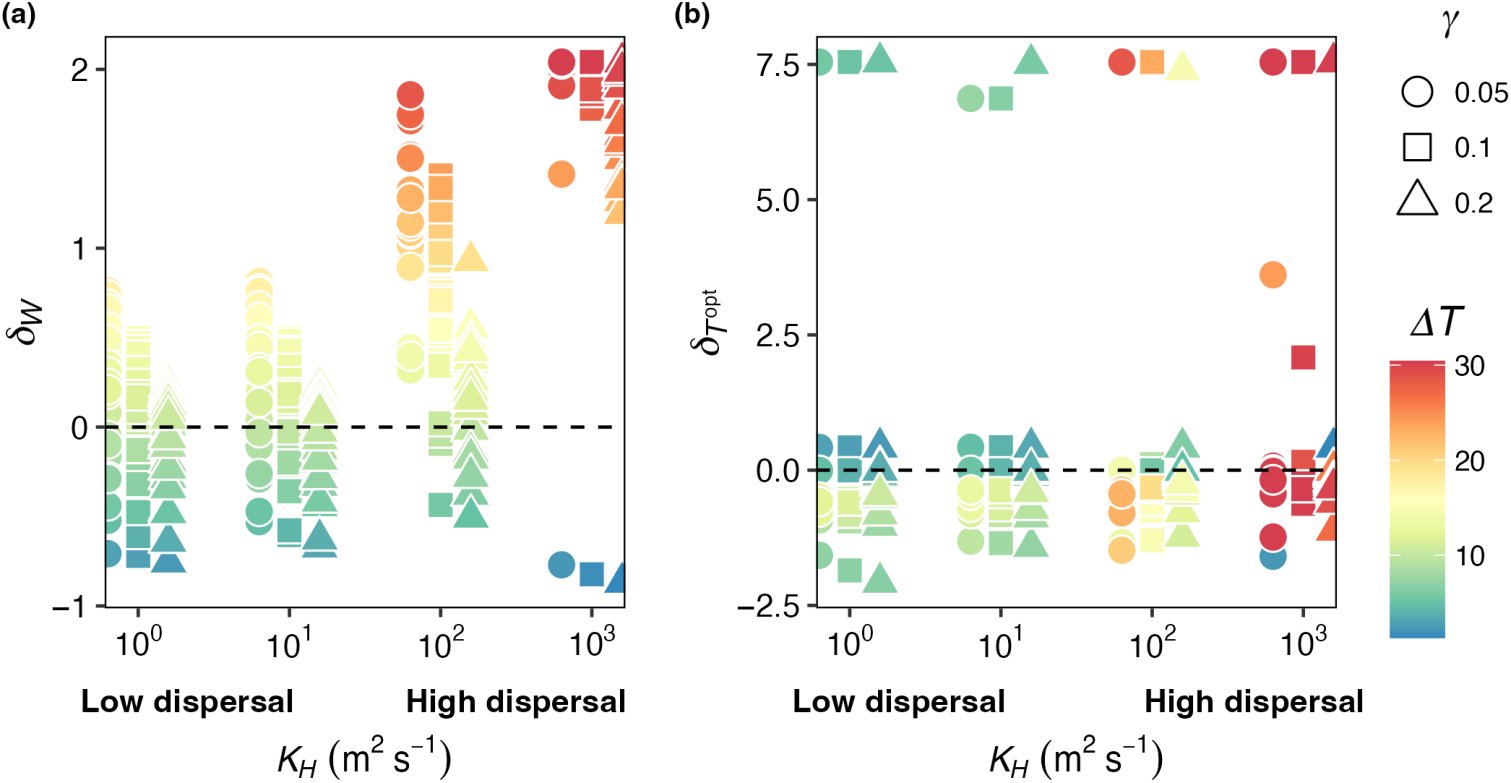
E4. (a) $\delta_{W}$ and (b) $\delta_{T^{\mathrm{opt}}}$ as function of temperature amplitude (°C) and dispersal magnitude ranging from low dispersal 10^0^ m^2^ s^−1^ to high dispersal 10^3^ m^2^ s^−1^ for low (circles; 0.05), medium (squares; 0.1), and high (triangles; 0.2) phytoplankton mortality scaling constants (γ). $\delta_{W}$ is the difference between realized and fundamental niche widths, divided by the fundamental niche width (Equation 9). $\delta_{T^{\mathrm{opt}}}$ is the difference between realized and fundamental temperature optimums, divided by the fundamental temperature optimum (Equation 10). Points are colored by the full range of temperatures an organism experienced across the whole domain in the last 5 years of a 50‐year integration of the model.

Across all mortality scaling factors (γ = 0.05, 0.1, and 0.2), realized temperature niche widths (*W*_*R*_) increased with increasing seasonal temperature variability and increasing dispersal magnitude (Figure 12a). When dispersal magnitude was highest (*K*_*H*_ = 10^3^ m^2^ s^−1^), almost all realized temperature niche widths were wider than the fundamental temperature niche widths (*δ*_*W*_ > 0; Figure 12a). When dispersal magnitude was lower (i.e., *K*_*H*_ = 10^0^ m^2^ s^−1^), realized temperature niche widths were wider than fundamental temperature niche widths when temperature amplitude was higher. Low dispersal magnitude combined with low temperature variability resulted in realized temperature niche widths that were narrower than the fundamental temperature niches (*δ*_*W*_ < 0; cooler colors in Figure 12a). For any given dispersal magnitude, the maximum *δ*_*W*_ decreased as mortality increased except for when dispersal was highest where some species were able to disperse the full range of modeled temperatures (Figure 12a). As dispersal magnitude and temperature variability increased, there was greater variability in the realized optimal temperature across species, but overall, there was no effect of dispersal, temperature variability, or mortality scaling factor on realized temperature niches ($\delta_{T^{\mathrm{opt}}}$; Figure 12b). We still find, as in E2 (Figure 6b), that polar species with low *T*_opt_ values present high $\delta_{T^{\mathrm{opt}}}$ values due to only being advected into warmer waters.

**FIGURE 13:**
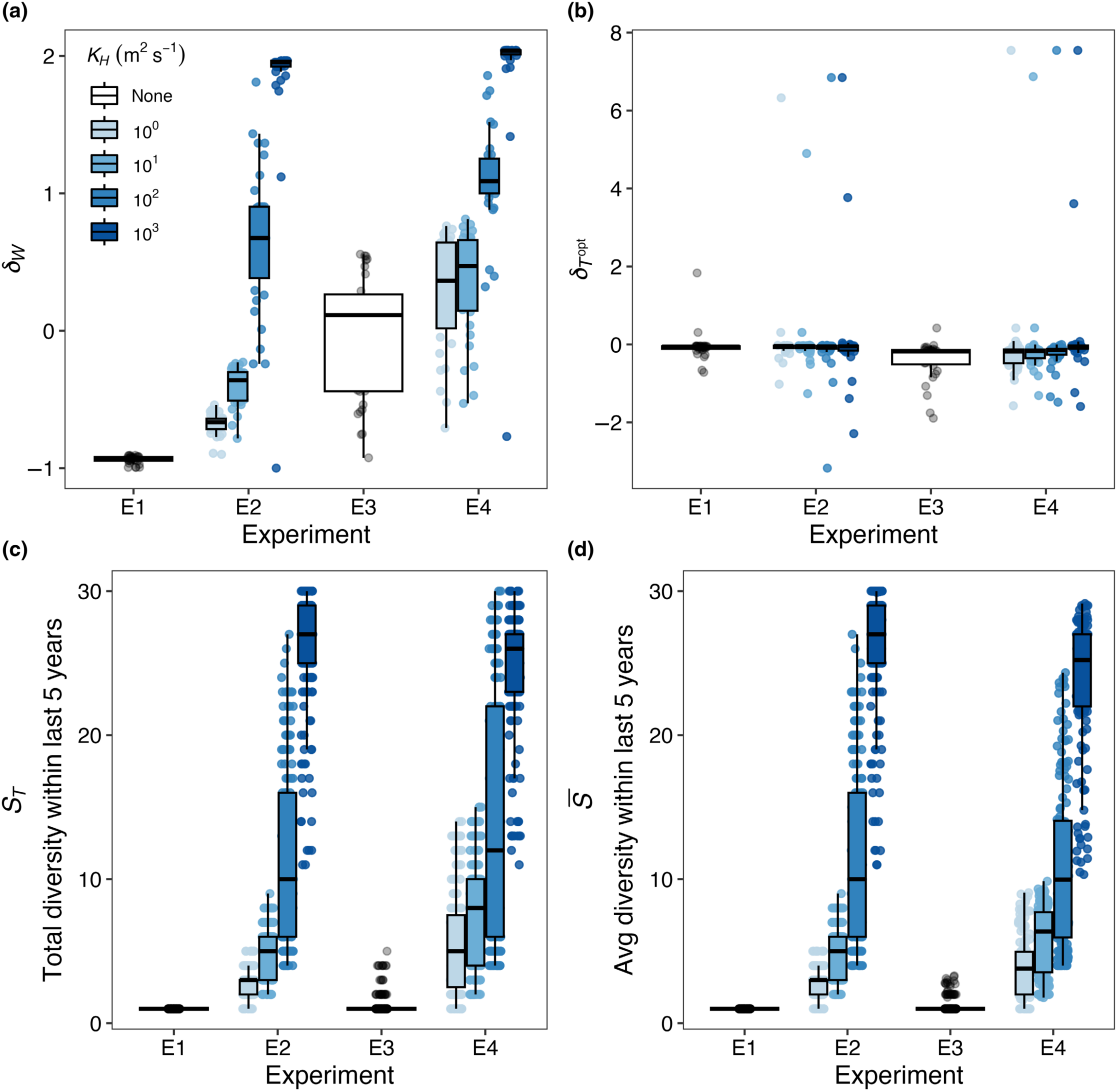
Comparison of (a) $\delta_{W}$, (b) $\delta_{T^{\mathrm{opt}}}$, (c) *S*_*T*_, and (d) $\bar{S}$ across all four experiments—E1: control; E2: spatial mass effects only; E3: temporal storage effects only; and E4: combined effects. Points for each experiment in panels a to b represent niche values for each surviving species and points in panels c to d represent diversity metric values within each box. Box plots show the 25th and 75th percentile (bottom and top edges), the 50th percentile (middle line), and the vertical lines are ±1.5 times the interquartile range, estimating the 95% confidence interval. Colors represent the different dispersal magnitudes that we tested ranging from low dispersal (10^0^; light blue) to high dispersal (10^3^; dark blue). Boxes in E1 and E3 are not colored because there was no dispersal between boxes. Points are jittered along the x‐axis for easier visualization. $\delta_{W}$ is the difference between realized and fundamental niche widths, divided by the fundamental niche width (Equation 9). $\delta_{T^{\mathrm{opt}}}$ is the difference between realized and fundamental temperature optimums, divided by the fundamental temperature optimum (Equation 10). $\bar{S}$ is the average number of species present at any point during the last 5 years of a 50‐year integration of the model, while *S*_*T*_ is the total number of species present during the last 5 years.

Figure 13 shows a summary of how niche widths (*δ*_*W*_), temperature niche optimums ($\delta_{T^{\mathrm{opt}}}$), average diversity ($\bar{S}$), and total diversity (*S*_*T*_) vary across Experiments 1–4 (E1–E4) for a range of dispersal strengths. Realized temperature niche widths (Figure 13a) increased with increasing dispersal strength and temporal temperature variation. Realized temperature niche optimums, however, were not strongly affected by either dispersal or temporal temperature variability (Figure 13b). Both total (Figure 13c) and average diversity (Figure 13d) increased with increasing dispersal strength and temperature variability.

## 4 DISCUSSION

Using a simple metacommunity model, we found that increasing dispersal and seasonal temperature variability increased realized niche widths and community diversity but did not affect realized temperature optimums for growth. Here, we discuss temporal storage effects, spatial mass effects, and source‐sink dynamics in the model, and how simplifications of the model guide our interpretations of the results.

### 4.1 Temporal storage effects

When temperature was constant with no dispersal (Figure 3), the model species with a fundamental optimum temperature for growth ($T_{F}^{\mathrm{opt}}$) closest to the constant temperature of the box outcompeted all others (Experiment 1, or E1; Figure 3). Similarly, in Experiment 3 (E3), when temperature amplitude was low with no dispersal (Figure 7), the model species with a fundamental optimum temperature for growth ($T_{F}^{\mathrm{opt}}$) closest to the mean temperature of the box outcompeted all others. However, in E3 and E4, we found that as seasonal temperature amplitude increased (Figures 8 and 11), regardless of dispersal between boxes, model diversity ($\bar{S}$ and *S*_*T*_) and realized temperature niche widths increased ($\delta_{W}$), although there was no effect on the difference between fundamental ($T_{F}^{\mathrm{opt}}$) and realized optimum temperatures ($T_{R}^{\mathrm{opt}}$) for growth ($\delta_{T^{\mathrm{opt}}}$; Figures 9 and 12). These model results, not only illustrated most clearly in Experiment 3 (E3) but also seen in E4, are caused by a temporal storage effect linked to the seasonal changes in temperature, as has been studied previously (Chesson, 2000; Descamps‐Julien & Gonzalez, 2005; Kremer & Klausmeier, 2017; Scranton & Vasseur, 2016). The changing temperature allowed for a temporal succession of model species with different temperature optimums for growth, and because model species persisted beyond when their temperature‐dependent specific growth rate is optimum, in many cases more than one model species existed at the same time (allowing for higher $\bar{S}$). The temporal succession facilitated a greater number of species present at some point over the year also (higher *S*_*T*_). The temporal storage effect also caused modeled realized temperature niches within a single model box to be wider than fundamental temperature niches, because species were able to persist well outside their ideal thermal conditions for growth, either due to a weakly positive net growth rate or a long, slow decline from high abundance conditions during a model “bloom.” However, temporal storage effects were weakened by increases in mortality (Figures 8 and 9). When the strength of mortality increased, model species abundance decreased quickly when mortality exceeded growth, and persistence outside of ideal thermal conditions was weaker. In other words, the strength of mortality of model microbes, caused by grazing, viruses, or any other source, is determined in large part by the strength of the temporal storage effect in the model.

In contrast, we found no evidence in the model for the temporal storage effect influencing realized optimum temperatures ($T_{R}^{\mathrm{opt}}$; Figure 9b). The species in our model did, however, have realized niche optima that were, on average, slightly colder than the fundamental temperature niche optimums ($\delta_{T_{\mathrm{opt}}}<0$; Figure 9b). Previous studies (Kingsolver et al., 2013; Smith et al., 2021) have observed this pattern across a range of organisms. Fundamental temperature niches are typically, and in this model, assumed to have a left‐ or negatively‐skewed curve where growth above the optimum temperature decreases rapidly compared to growth below the optimum, as measured from laboratory experiments where growth is calculated from incubations at constant temperatures (Anderson et al., 2021; Norberg, 2004; Thomas et al., 2012). Jensen's Inequality suggests that, in nonlinear systems, time‐averaged growth under variable conditions differs from growth under average conditions (Bernhardt et al., 2018). In our model, phytoplankton growth decelerates with temperature (i.e., the second derivative of the thermal response curve is negative), leading to realized temperature niche optima that are colder than fundamental temperature optima.

### 4.2 Spatial mass effects

We found that when temperature is constant (E2), increasing dispersal strength increased model diversity within each location ($\bar{S}$ and *S*_*T*_; Figure 5) and increased realized temperature niche widths (*W*_*R*_) of model species compared to their fundamental niches ($\delta_{W}$; Figure 6a), although there was no effect on realized optimum temperature for growth ($T_{F}^{\mathrm{opt}}$) for model species compared to their fundamental niches ($\delta_{T_{\mathrm{opt}}}$; Figures 6b). Phytoplankton community composition and dynamics are not only influenced by local environmental conditions and ecological processes but also by immigration and emigration from and to other locations (Hellweger et al., 2014; Jönsson & Watson, 2016; Leibold et al., 2004; Ward et al., 2021). Spatial mass effects describe the physical displacement of species across spatially separated patches (Leibold, 1997; Leibold et al., 2004; Shoemaker & Melbourne, 2016; Steiner & Leibold, 2004). When the rates of dispersal were very low in the model, species sorting dominated ecological outcomes (Figures 4, 5, 6). As the rate of dispersal increased, dispersal reintroduced species faster than competition removed them, such that overall diversity ($\bar{S}$ and *S*_*T*_; Figure 5) was higher than in the low‐dispersal case (Leibold et al., 2004; Shoemaker & Melbourne, 2016). In addition to an increase in diversity, we found that increasing dispersal magnitude, with or without the addition of temporal variability, increased realized niche widths (*W*_*R*_) compared to their fundamental niches ($\delta_{W}$; Figure 6a). At the highest rates of dispersal, realized niche widths were wider than fundamental niche widths ($\delta_{W}$ > 0; Figure 6a), illustrating how spatial mass effects can rescue or buffer species from local extirpation. In regions of the ocean with high dispersal, spatial mass effects could be driving a large portion of the observed diversity, and species are likely to be present in the community even though they may have low or negative net growth there (e.g., Barton et al., 2010; Clayton et al., 2013). For example, over a matter of days, *Prochlorococcus* in the Gulf Stream can be moved hundreds of kilometers and ultimately encounter conditions outside their expected thermal tolerance (Cavender‐Bares et al., 2001). Recent field‐studies have confirmed that the composition of marine microbial communities is strongly impacted by dispersal, not just local environmental conditions (Villarino et al., 2022).

We found no clear relationship between dispersal strength and realized optimum temperatures for growth in the model ($T_{F}^{\mathrm{opt}}$; Figure 6b). This was likely because dispersal in the model was equal in all directions, such that changing the model dispersal rates did not appreciably change the realized optimum temperatures for growth for each model species.

The effects of dispersal on diversity ($\bar{S}$ and *S*_*T*_) and realized niche widths ($\delta_{W}$) were dampened as mortality increased (Figures 5 and 6). For the same dispersal magnitude, we found less diversity and narrower realized temperature niches with increasing mortality. Lower model mortality rates allow for model organisms to spread and persist further from their source, whereas higher mortality rates tend to minimize the ecological significance of spatial mass effects on model phytoplankton assemblages.

### 4.3 Source‐sink dynamics

Source‐sink dynamics are common in ecological communities connected via dispersal (Gonzalez & Holt, 2002; Holt, 1985; Holt et al., 2003; Leibold et al., 2004; Roy et al., 2005). “Source” populations occur where conditions are favorable for the population to exist, and “sink” populations occur where they would not persist without dispersal from other locations (the rescue effect). These source‐sink dynamics control model dynamics, which we discuss further there.

**FIGURE 14:**
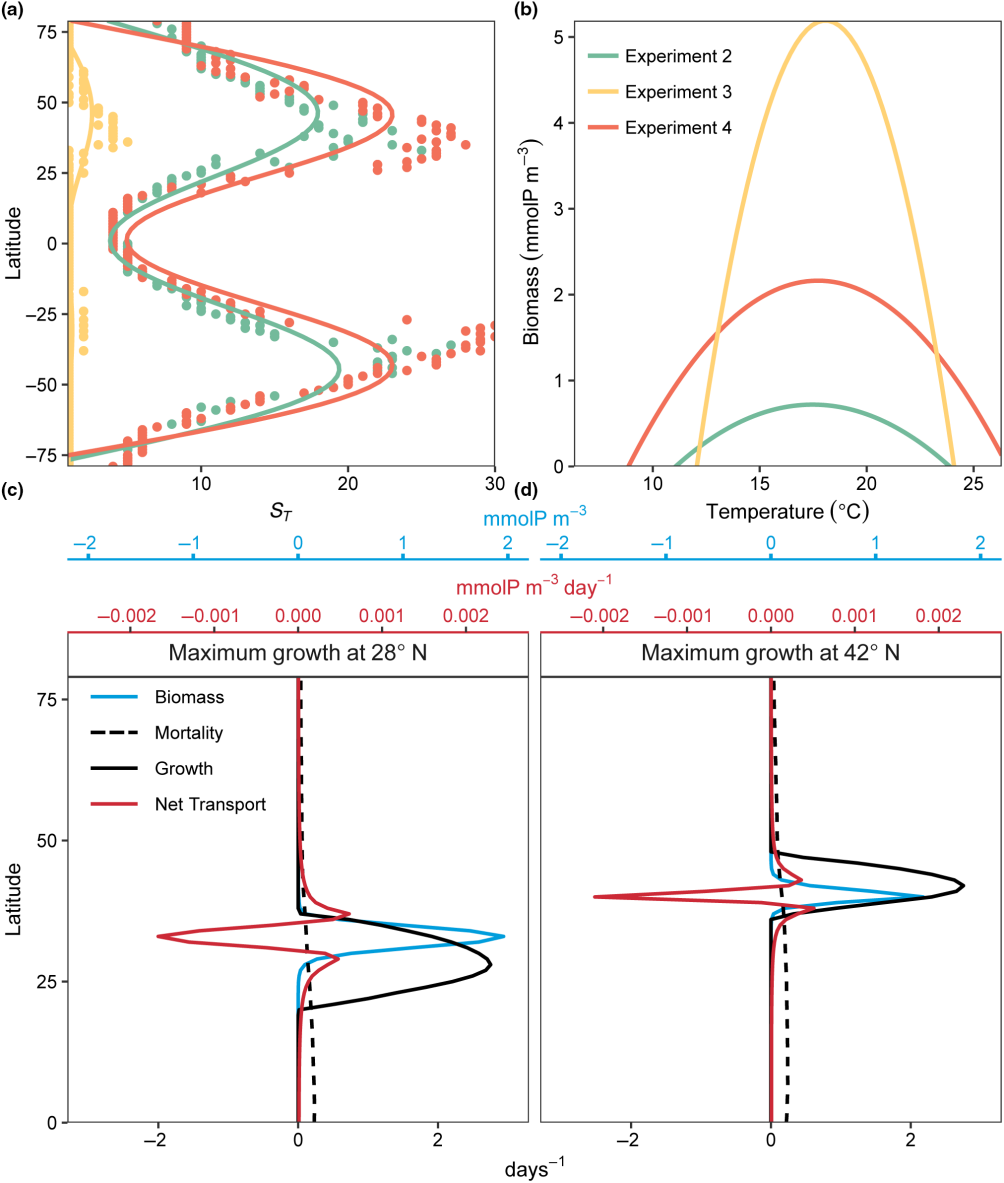
(a) Total diversity present during the last 5 years of a 50‐year integration of the model (*S*_*T*_) across all latitudes under three experiments with low mortality scaling (γ = 0.05; see Figure 2)—Experiment 2 (E2): dispersal equal to *K*_*H*_ × 10^2^ m^2^ s^−1^ with constant temperature (green); Experiment 3 (E3): natural temperature variability with no dispersal (yellow); and Experiment 4 (E4): dispersal equal to *K*_*H*_ × 10^2^ m^2^ s^−1^ with natural temperature variability (orange). (b) Realized temperature niches for a species with an optimum temperature of 19°C under the same three experiments as in the panel a. (c–d) Instantaneous model dynamics for the same species in the panel (b) with an optimal temperature of 19°C at the time that the growth rate is maximum at 28° N (c) and 42° N (d). Growth maxima occurred during Winter at 28° N and during Summer at 42° N. The solid black line is temperature‐dependent specific growth rate (μ(*T*); days^−1^); the dashed black line is temperature‐dependent mortality rate (*m*(*T*) = $ae^{\mathrm{bT}}$; days^−1^); the solid red line is net transport (τ; mmolP m^−3^ days^−1^; top red axes); and the solid blue line is biomass in each model box (*P*; mmolP m^−3^; top blue axes). Negative net transport means the location is a source of biomass, while positive net transport means the location is a sink for biomass.

Spatial and temporal mass effects, independently, increased model diversity and realized niche widths (E2 in Figures 5 and 6 and E3 in Figures 8 and 9). The model indicated that diversity ($\bar{S}$ and *S*_*T*_) was higher when the temporal and spatial mass effects were combined (E4), particularly in regions where temperature variability was high (Figure 14a). The model also illustrated how certain areas where an organism has high fitness and biomass can serve as a source of biomass for adjacent areas where the same organism's fitness is relatively low. These source‐sink dynamics underpin the widening of the realized temperature niche relative to the fundamental niche ($\delta_{W}$) when temporal and spatial mass effects were combined (in this case for just one illustrative model species with an optimum temperature for growth of 19°C; Figure 14b). The source location occurs where the fitness of a particular organism is relatively high, and the sink is where the population of that organism is sustained by dispersal, but these source and sink locations change over the year. For example, consider again the model phytoplankton with an optimum temperature for growth of 19°C (Figure 14c,d). In February and August, respectively, its biomass (blue lines) is maximum at 28° N and 42° N. The actual growth rate at these moments (black lines) did not precisely coincide latitudinally with biomass peaks because of temporal lags between maximum growth rate and biomass. The areas of high biomass had negative net transport, meaning they acted as a source of biomass for adjacent areas. These adjacent areas were a sink of biomass where local fitness was relatively low and the population was sustained by dispersal from other areas. Thus, in marine settings, a species may be present in space and time even when its fitness is relatively low, due to either or both temporal storage and spatial mass effects, provided that the rates of mortality are sufficiently low to allow for temporal persistence and spatial dispersal of organisms.

### 4.4 Model simplifications and their implications

We created a simple metacommunity to study how spatial mass and temporal storage effects shape realized temperature niches and community diversity. However, given the idealized nature of the model, we did not expect model species distributions or diversity gradients to closely match observed, global‐scale patterns. Here, we briefly discuss key model simplifications and how the model simplifications in traits, trophic relationships, ocean circulation, mutations, and stochasticity make direct comparison with ocean observations challenging.

Temperature variability in this model was simplified from observations to create a repeating and smooth seasonal cycle within a given 1° latitude band averaged across all longitudes. Thus, temperature variations occurring on higher (e.g., internal waves, storms, and upwelling events) and lower frequencies (e.g., interannual variations and anthropogenic climate change) were not considered. Environmental variations at these unrepresented scales clearly influence community structure and competitive outcomes (Barton et al., 2020; Vasseur et al., 2014). In addition, all species in the model were seeded with the same fundamental niche width (10°C), which we based upon the average of a range of observed niche widths in the North Atlantic (Irwin et al., 2012). This choice ignores real variations in niche widths and their associated hypothetical trade‐offs, such as among temperature generalists and specialists (Kingsolver, 2009).

Additionally, the model did not resolve important trait variations, such as cell size, nutrient uptake affinity, and nutrient storage, and neither did the model explicitly resolve losses to zooplankton grazing, viral lysis, or other factors. There are trade‐offs between competitive traits for nutrient acquisition, cell size, and light availability that shape an organism's ecological niche (Edwards et al., 2012, 2013; Litchman et al., 2012). For simplicity, however, we ignored these important ecological dimensions to focus on the univariate temperature niche. These omitted traits mean, for example, that the model dynamics do not accurately represent seasonal depletion of nutrients due to phytoplankton blooms (e.g., Edwards et al., 2012) or biogeographic and diversity patterns tied to nutrients, light, or other factors (e.g., James et al., 2022). Phytoplankton mortality in the model increased exponentially with temperature (Equation 4), using the same exponents *a* and *b* as reported for the temperature sensitivity of growth (e.g., Equation 3). However, while this simplification was desirable in order to have growth and mortality roughly matched across a wide range of temperatures for model phytoplankton, recent studies have shown that the temperature dependence of mortality may differ from growth (e.g., Baker & Geider, 2021; Demory et al., 2017).

Our model utilized isotropic dispersal but ocean currents are much more dynamic both temporally and spatially. More realistic patterns of dispersal including, for example, wind‐driven currents such as the Gulf Stream, may produce more plausible source and sink areas for microbial populations (e.g., Ward et al., 2021) and hotspots of diversity where adjacent communities mix together (e.g., Clayton et al., 2013).

The model did not represent mutations or demographic stochasticity, although these processes play important roles in natural systems. Selection on new mutations and existing intraspecific variability can lead to changes in species niches over time (Collins et al., 2014; Lohbeck et al., 2012). Our model included just one phenotype per model species, defined by its temperature niche, that was able to persist in some cases in suboptimal growth conditions due to spatial mass and temporal storage effects. However, marine phytoplankton species often have considerable standing genetic variation (e.g., Biller et al., 2015), which widens the fundamental and realized niche for that species (Smith et al., 2021). Some of this standing genetic diversity may be maintained by dispersal and temporal environmental variation. The model also did not include demographic stochasticity (Lande, 1993; Shoemaker et al., 2020), which is critically important for dynamics of small populations in particular. Ward et al. (2021) found that demographic stochasticity did not significantly affect microbial populations where they were abundant, for example, in their core ranges, but did increase the chance of local extinction when microbial populations were very small. As such, our model is optimized for studying microbial dispersal between nearby regions and persistence through time, rather than through strong selection gradients (e.g., a cold water‐adapted cell passing through the equatorial zone) that dramatically lower population abundance. Historical contingencies and priority effects (e.g., Sefbom et al., 2015) are therefore not resolved in our model.

## 5 CONCLUSION

Our original motivation for undertaking this modeling study was to better understand how and why realized temperature niches for the marine cyanobacterium *Prochlorococcus* are wider than fundamental temperature niches. In the model, temporal storage and spatial mass effects generated increased diversity and realized temperature niche widths. However, the combined effects created realized temperature niches that exceeded the fundamental temperature niches and further increased diversity. This model was idealized but provided a useful framework for asking how physical processes such as temperature variability and dispersal shape phytoplankton realized temperature niches. Much of the research focusing on microbial diversity in the oceans so far has neglected the roles that spatial mass and temporal storage effects may play in shaping diversity and biography, and our model helps illustrate that these processes may be important under certain ocean conditions. For example, the seasonal temporal storage effects are likely to be strongest in regions with strong seasonal variations in temperature, such as mid‐latitude and coastal ocean regions. Because the strength of temporal storage effects decreased with increasing mortality rates in the model, the ecological importance of temporal storage effects may be heightened specifically during winter and spring when predators are relatively scarce due to overwintering (Mauchline, 1998) or dilution by deep mixed layers (Behrenfeld & Boss, 2014). Spatial mass effects are likely strongest where horizontal advection and mixing are highest, such as western boundary currents. Like temporal storage effects, the ecological importance of spatial mass effects may be highest when rates of phytoplankton mortality are lowest. While further observational and modeling work can constrain the roles that temporal storage and spatial mass effects play in setting distributions of *Prochlorococcus* ecotypes, our model suggested that these mechanisms are likely to be influential for the ecology of these and other microbial taxa.

Beyond just understanding the distribution of species in the ocean, these results have direct implications for species distribution modeling. Species distribution models, or SDMs, are often used to predict temporal and spatial distributions of species based upon (usually limited) data describing the realized niche of a particular species and more widespread data describing environmental conditions (Elith & Leathwick, 2009). Such models are increasingly used to understand patterns of biogeography in marine plankton, and how they may change in response to climate warming (e.g., Barton et al., 2016; Brun et al., 2015; McGinty et al., 2021). The influence of temporal storage and spatial mass effects on realized niches, and the high likelihood that the ecological impact of these processes change in space and time, represent yet another challenge for applying species distribution models to make biogeographic and ecological projections in response to climate change.

Finally, this simple model highlights how two fundamental processes acting ubiquitously in the ocean—environmental and population change through time and dispersal of organisms—play an important and often overlooked role in shaping marine microbial spatial and temporal patterns of distribution, realized niches, and community diversity.

## AUTHOR CONTRIBUTIONS

**Alaina N. Smith:** Conceptualization (equal); formal analysis (lead); investigation (lead); methodology (lead); visualization (lead); writing – original draft (lead); writing – review and editing (equal). **Andrew D. Barton:** Conceptualization (equal); supervision (lead); writing – review and editing (equal).

## CONFLICT OF INTEREST STATEMENT

The authors have no conflict of interest to declare.

## Supporting information


Data S1.

## Data Availability

All figures and analysis were created through model simulations and the code is available on GitHub at https://github.com/anoelsm/temperature‐niche‐model.
