# Supplementary material for: Effects of dispersal and temperature variability on phytoplankton realized temperature niches
Source: Ecol Evol. 2024 Feb 7;14(2):e10882. doi: 10.1002/ece3.10882 (PMC10847892; doi:10.1002/ece3.10882)
Supplement: Supplementary file 1 — Data S1. [file ECE3-14-e10882-s001.pdf]

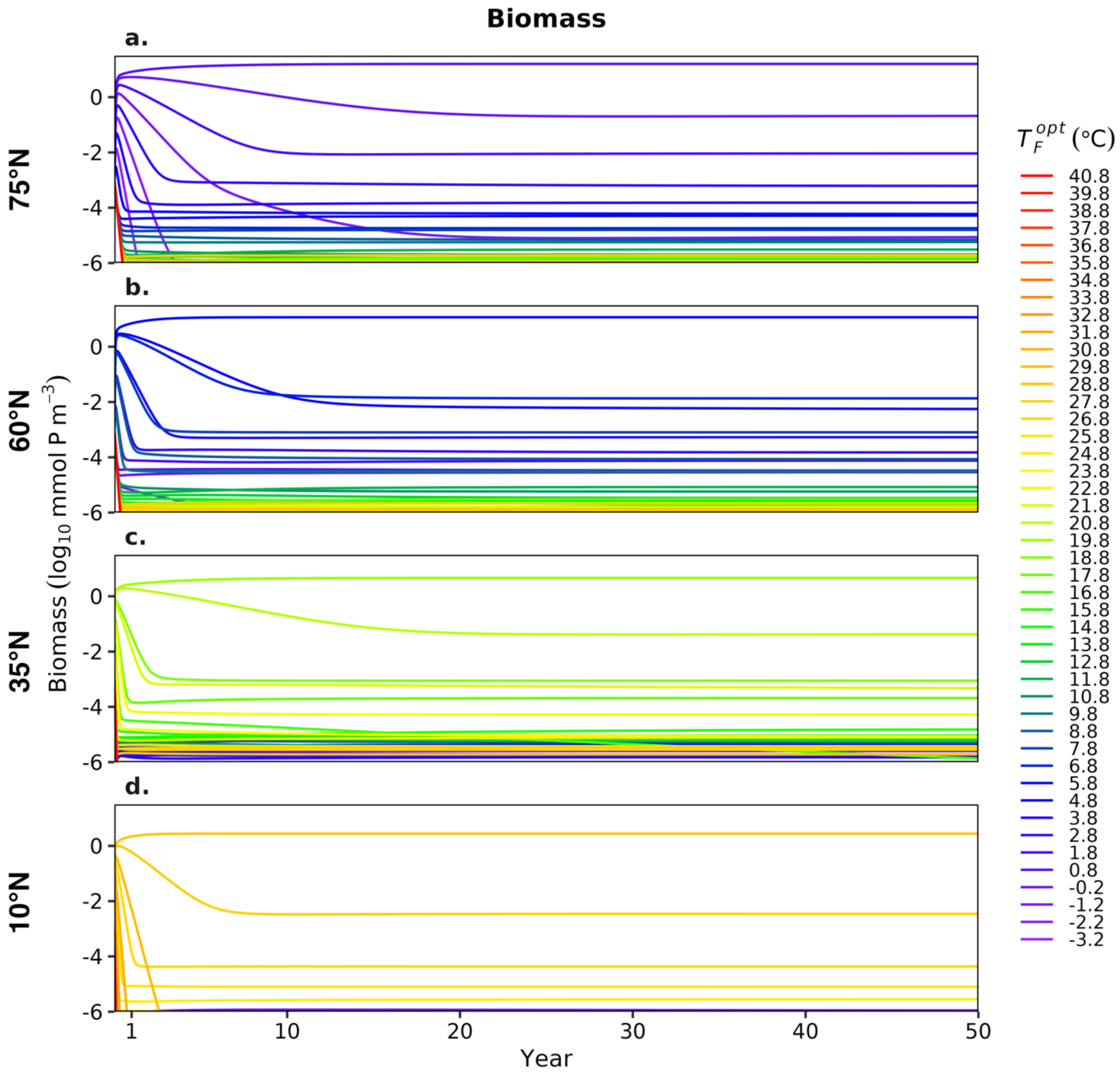

Figure S1. ***E1.*** Biomass for surviving phytoplankton species in four illustrative model boxes (rows) ranging from colder high latitudes (top) to warmer low-latitudes (bottom) over a 50-year model integration. The four illustrative model boxes represented areas centered on 75ºN (a), 60ºN (b), 35ºN (c), and 10ºN (d), as indicated in Fig. 1c. Species are colored by their corresponding optimal temperature (T_F^{opt}) values. There was no dispersal between boxes, temperature was constant in each box, and mortality scaling constant (γ) was 0.05. Fig. 3 presents the last 5 years this model integration.

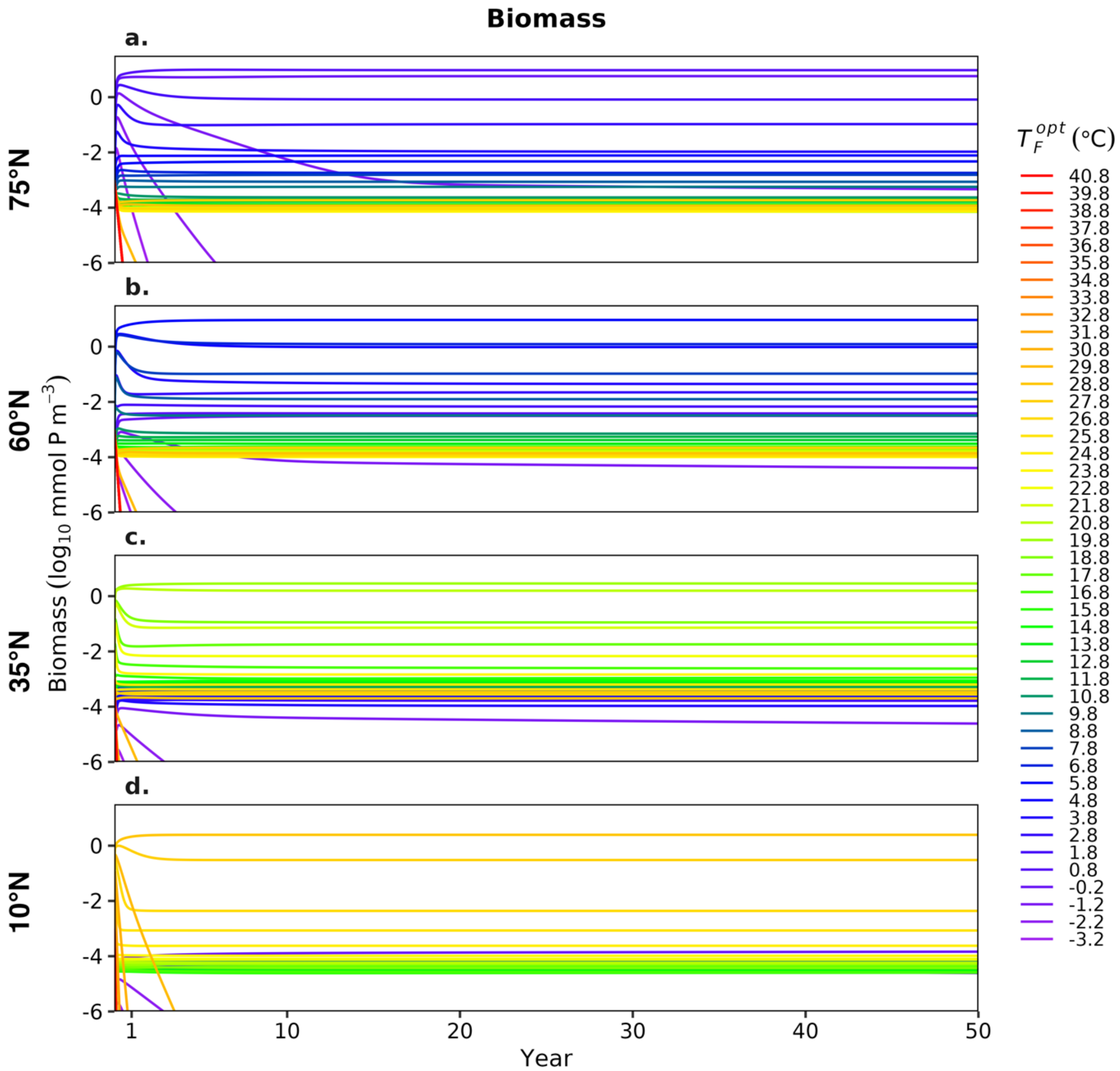

Figure S2. ***E2.*** Biomass for surviving phytoplankton species in four illustrative model boxes (rows) ranging from colder high latitudes (top) to warmer low-latitudes (bottom) over a 50-year model integration. The four illustrative model boxes represented areas centered on 75ºN (a), 60ºN (b), 35ºN (c), and 10ºN (d), as indicated in Fig. 1c. Species are colored by their corresponding optimal temperature (T_F^{opt}) values. Dispersal magnitude (K_H) for this model run was 10^2 m^2 s^{-1}, temperature was constant in each box, and the mortality scaling constant (γ) was equal to 0.05. Fig. 4 presents the last 5 years of this model integration.

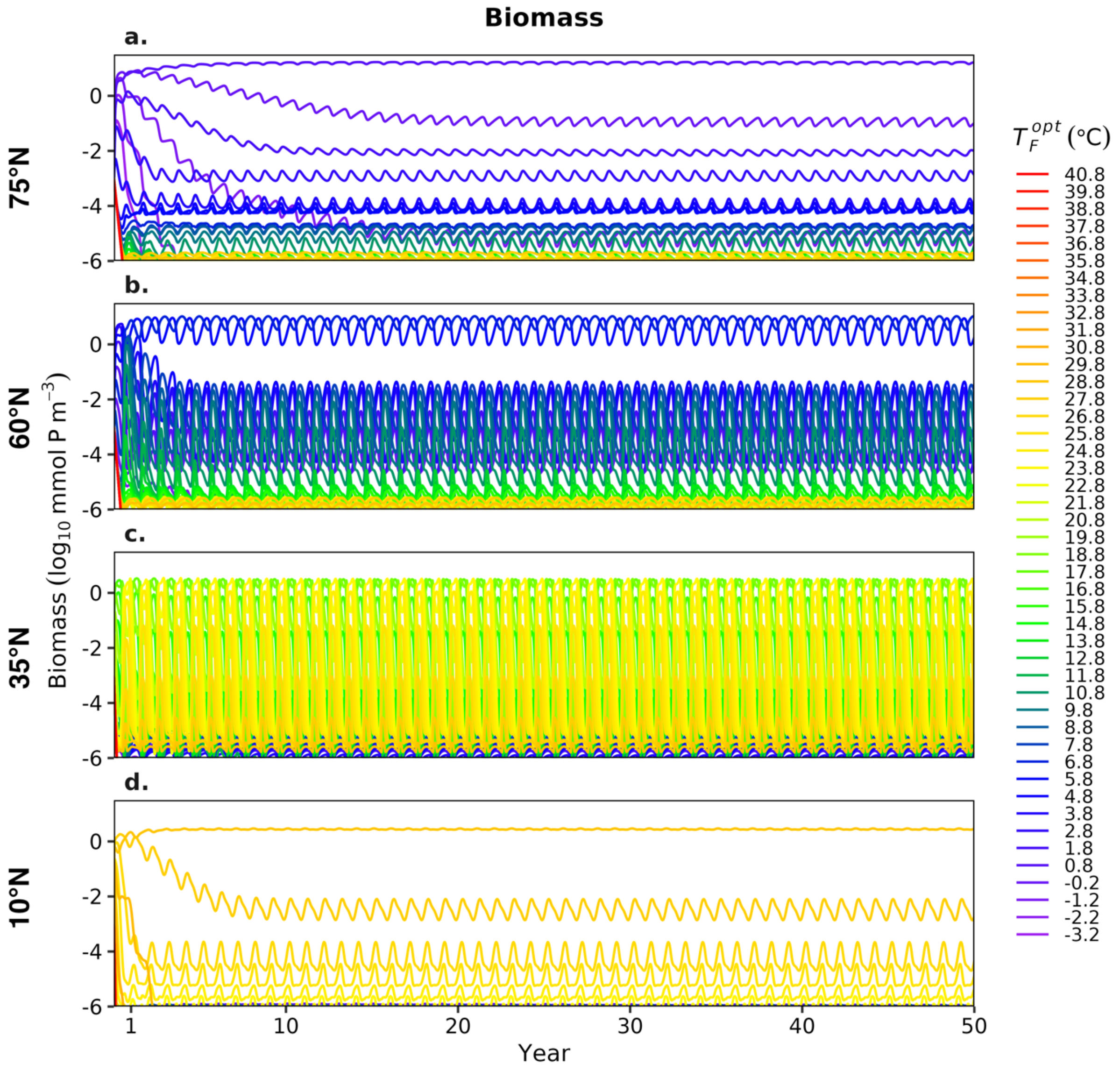

Figure S3. ***E3.*** Biomass for surviving phytoplankton species in four illustrative model boxes (rows) ranging from colder high latitudes (top) to warmer low-latitudes (bottom) over a 50-year model integration. The four illustrative model boxes represented areas centered on 75ºN (a), 60ºN (b), 35ºN (c), and 10ºN (d), as indicated in Fig. 1c. Species are colored by their corresponding optimal temperature (T_F^{opt}) values. Dispersal magnitude (K_H) for this model run was zero, temperature was allowed to vary naturally in each box, and the mortality scaling constant (γ) was equal to 0.05. Fig. 7 presents the last 5 years of this model integration.

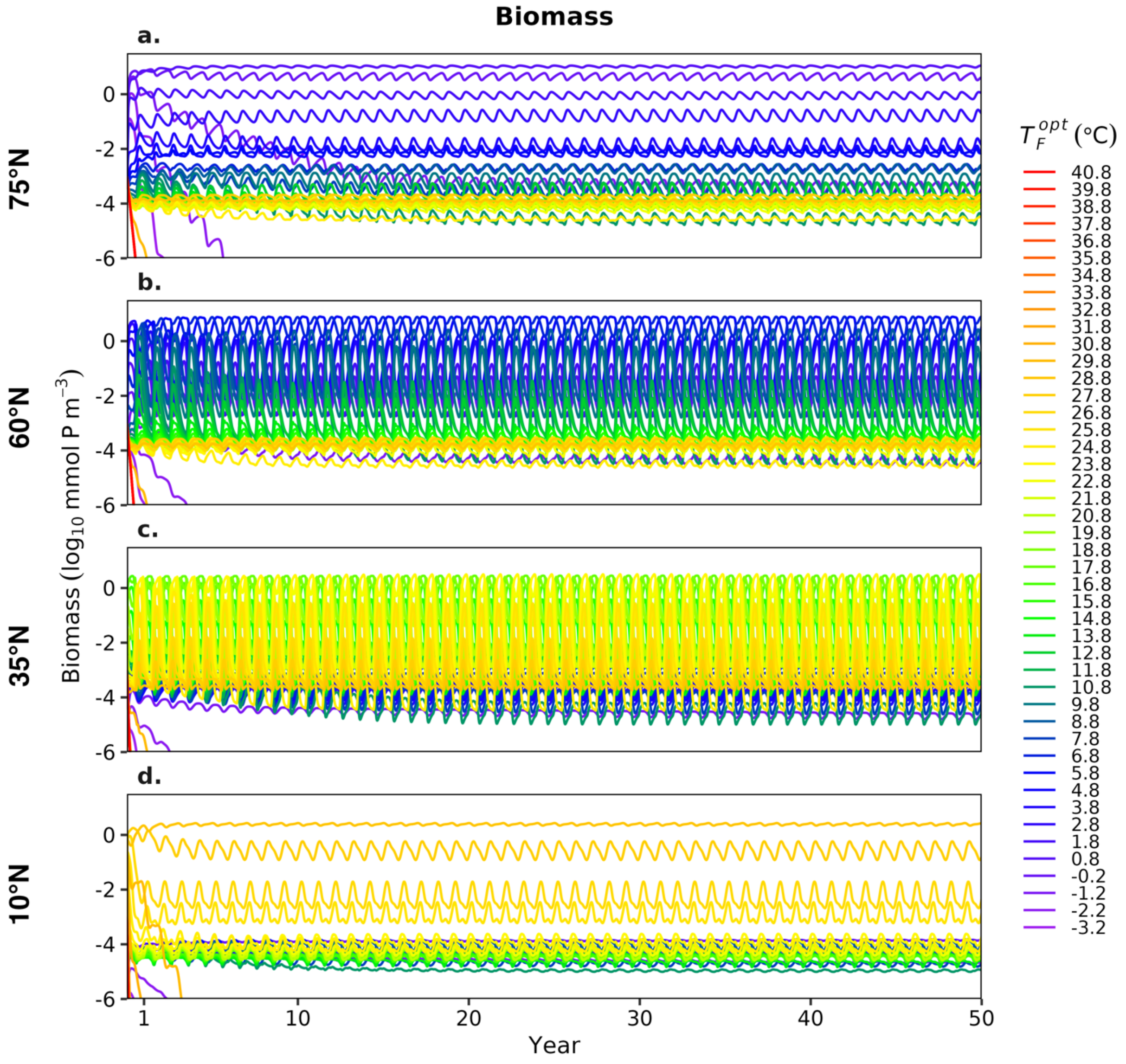

Figure S4. ***E4.*** Biomass for surviving phytoplankton species in four illustrative model boxes (rows) ranging from colder high latitudes (top) to warmer low-latitudes (bottom) over a 50-year model integration. The four illustrative model boxes represented areas centered on 75ºN (a), 60ºN (b), 35ºN (c), and 10ºN (d), as indicated in Fig. 1c. Species are colored by their corresponding optimal temperature (T_F^{opt}) values. Dispersal magnitude (K_H) for this model run was 10^2 m^2 s^{-1}, temperature was allowed to vary naturally in each box, and the mortality scaling constant (γ) was equal to 0.05. Fig. 10 presents the last 5 years of this model integration.
